# Artemisitene suppresses rheumatoid arthritis progression via modulating METTL3‐mediated N6‐methyladenosine modification of *ICAM2* mRNA in fibroblast‐like synoviocytes

**DOI:** 10.1002/ctm2.1148

**Published:** 2022-12-19

**Authors:** Jian Chen, Xian Lin, Juan He, Dandan Liu, Lianhua He, Miaomiao Zhang, Huijie Luan, Yiping Hu, Cheng Tao, Qingwen Wang

**Affiliations:** ^1^ Department of Rheumatism and Immunology Peking University Shenzhen Hospital Shenzhen Guangdong China; ^2^ Shenzhen Key Laboratory of Inflammatory and Immunology Diseases Shenzhen Guangdong China; ^3^ School of Basic Medical Science Guangzhou University of Chinese Medicine Guangzhou Guangdong China; ^4^ School of Pharmacy Guangdong Medical University Dongguan Guangdong China

**Keywords:** artemisitene, fibroblast‐like synoviocytes, ICAM2, METTL3, rheumatoid arthritis

## Abstract

**Background:**

Rheumatoid arthritis (RA) is a chronic autoimmune disease. We previously revealed that the natural compound artemisitene (ATT) exhibits excellent broad anticancer activities without toxicity on normal tissues. Nevertheless, the effect of ATT on RA is undiscovered. Herein, we aim to study the effect and potential mechanism of ATT on RA management.

**Methods:**

A collagen‐induced arthritis (CIA) mouse model was employed to confirm the anti‐RA potential of ATT. Cell Counting Kit‐8 (CCK‐8) and 5‐ethynyl‐2'‐deoxyuridine (EdU) assays, cell cycle and apoptosis analysis, immunofluorescence, migration and invasion assays, quantitative real‐time PCR (RT‐qPCR), Western blot, RNA‐sequencing (RNA‐seq) analysis, plasmid construction and lentivirus infection, and methylated RNA immunoprecipitation and chromatin immunoprecipitation assays, were carried out to confirm the effect and potential mechanism of ATT on RA management.

**Results:**

ATT relieved CIA in mice. ATT inhibited proliferation and induced apoptosis of RA‐fibroblast‐like synoviocytes (FLSs). ATT restrained RA‐FLSs migration and invasion via suppressing epithelial–mesenchymal transition. RNA‐sequencing analysis and bioinformatics analysis identified intercellular adhesion molecule 2 (ICAM2) as a promoter of RA progression in RA‐FLSs. ATT inhibits RA progression by suppressing ICAM2/phosphoinositide 3‐kinase (PI3K)/protein kinase B (AKT)/p300 pathway in RA‐FLSs. Moreover, ATT inhibited methyltransferase‐like 3 (METTL3)‐mediated N6‐methyladenosine methylation of *ICAM2* mRNA in RA‐FLSs. Interestingly, p300 directly facilitated METTL3 transcription, which could be restrained by ATT in RA‐FLSs. Importantly, METTL3, ICAM2 and p300 expressions in synovium tissues of RA patients were related to clinical characteristics and therapy response.

**Conclusions:**

We provided strong evidence that ATT has therapeutic potential for RA management by suppressing proliferation, migration and invasion, in addition to inducing apoptosis of RA‐FLSs through modulating METTL3/ICAM2/PI3K/AKT/p300 feedback loop, supplying the fundamental basis for the clinical application of ATT in RA therapy. Moreover, METTL3, ICAM2 and p300 might serve as biomarkers for the therapy response of RA patients.

## Graphical abstract

1. ATT modulates METTL3/ICAM2/PI3K/AKT/p300 feedback loop in RA‐FLSs to manage RA.
2. METTL3 mediates m6A methylation of *ICAM2* mRNA and activates PI3K/AKT/p300 in RA‐FLSs.
3. ATT suppresses proliferation, migration and invasion and induces apoptosis in RA‐FLSs.

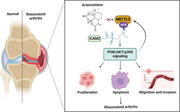

## 1 INTRODUCTION

Rheumatoid arthritis (RA) is a systematic autoimmune disease characterised by chronic synovitis and joint destruction.^1^ At present, the mechanisms concerning the initiation and progression of RA are undetermined. Fibroblast‐like synoviocytes (FLSs) are effector cells of synovial hyperplasia of RA and are regarded as tumour‐like cells harbouring biological characteristics such as abnormal proliferation, migration and invasion, resistance to apoptosis, and so on.^1^ Recently, increasing studies have proved that promoting apoptosis or inhibiting proliferation, migration and invasion of FLSs could efficiently prevent RA occurrence and development.^2^, ^3^, ^4^ Hence, seeking new drugs that suppress the pathological functions of RA‐FLSs has become a possible method for RA therapy.

In the past several decades, researchers have made many efforts to identify clinical biomarkers for the early diagnosis and prognosis of RA, which play an important role in new drug discovery.^5^ For example, Cheng et al.^6^ found GZMA, PRC1 and TTK as possible biomarkers for early RA diagnosis and therapeutic targets. In addition, the 14‐3‐3η protein was revealed to be a promising biomarker for the diagnostic, prognostic and therapeutic response of RA.^7^ Furthermore, it was reported that serum PGLYRP‑1 could be regarded as a biomarker for RA diagnosis.^8^ Intercellular adhesion molecule 2 (ICAM2), also known as CD102, is a class I transmembrane glycoprotein consisting of two immunoglobulin‐like C2 domains that bind to the protein molecules associated with lymphocyte functional antigen 1 (LFA‐1) on the surface of leukocytes.^9^ ICAM2 is involved in a variety of biological functions of numerous cell types, especially playing an important role in cell proliferation.^10^ Moreover, ICAM2 could mediate T‐cell‐induced phosphoinositide 3‐kinase (PI3K)/protein kinase B (AKT) activation and support cell survival.^11^ Nevertheless, the role of ICAM2 in RA is unclear.

RNA modifications present a novel level of posttranscriptional gene expression regulation and are involved in multiple biological activities, among which N6‐methyladenosine (m6A) modification is one of the most important forms of internal modifications in eukaryotic cells.^12^, ^13^ m6A modification is mainly mediated by m6A regulators, including methyltransferase, demethylase, and binding proteins (also recognised as ‘encoders’, ‘decoders’ and ‘readers’), to modulate RNA stability and translation efficiency.^14^ Recently, m6A modification has been reported to be essential for RA progression.^15^, ^16^ For example, methyltransferase‐like 3 (METTL3), an important methyltransferase for m6A modification is markedly increased in RA patients, and could attenuate the inflammatory response in lipopolysaccharide‐induced macrophages.^17^ Besides, recent research has demonstrated that the expression of YTHDF2, an m6A reader is decreased in RA patients’ peripheral blood mononuclear cells, and negatively correlated with erythrocyte sedimentation rate (ESR) level, C‐reactive protein (CRP) level, and so on.^18^ Moreover, our previous study also reported that the dysregulation of many m6A regulators, including METTL3, WTAP, YTHDF1, etc., affects RA progression.^19^ Therefore, targeting m6A regulators might be a feasible strategy for RA therapy.

Artemisinin is a well‐known antimalarial drug extracted from the plant *Artemisia annua* L. (compositae) with immunomodulatory effects, anti‐viral effects, anticancer activities, and so on.^20^, ^21^ Recently, artemisinin derivatives have been reported to have the potential of treating RA. For example, artesunate and dimeric artesunate phospholipid conjugates could relieve RA symptoms in RA animal models by immunomodulatory effects and related signalling pathways.^22^, ^23^ In addition, artesunate has anti‐proliferation, apoptosis‐ and autophagy‐inducing effects on chondrocytes of rat models with RA.^24^ Moreover, many studies have demonstrated that artesunate could regulate the physiological function of RA‐FLSs by inhibiting angiogenic factor expression,^25^ nuclear factor‐κB, and PI3K/AKT signalling pathway,^26^ as well as migration and invasion.^27^ Furthermore, Wang et al.^28^ reported that SM905 alleviates collagen‐induced arthritis (CIA) through suppression of inflammatory and Th17 responses. Artemisitene (ATT) is a dehydro analogue of artemisinin.^29^ Although ATT was first reported in the 1980s, little research about ATT has been reported so far. Chen and coworkers^30^, ^31^ reported that ATT induces the Nrf2‐dependent antioxidant response, and suppresses lung injury induced by bleomycin. Besides, ATT exhibits inhibitory activity on multiple cancers.^32^, ^33^ Our group recently demonstrated that ATT selectively eliminates human cancer cells by destabilising c‐Myc, resulting in DNA damage and apoptosis.^34^ Nevertheless, the effect of ATT on RA is undiscovered. Here, we aimed to assess the anti‐RA effect of ATT and further explore the molecular mechanism of ATT in controlling the pathological progress of RA, providing fundamental basis for the clinical application of ATT in RA therapy. Furthermore, the target proteins regulated by ATT in RA‐FLSs might serve as biomarkers for the early diagnosis or therapy response of RA patients.

## 2 MATERIALS AND METHODS

### 2.1 Animal models and drug administration

CIA mouse models were established in randomly assigned male DBA/1J mice purchased from SLAC Laboratory Animal Co., Ltd. (China) as we reported previously.^35^ On day 21, CIA mice were treated with solvent, methotrexate (MTX, 23 mg/kg; Yuanye Bio‐Technology Co., Ltd., China) or ATT (30 mg/kg; purity ≥98%, Toronto Research Chemicals, Canada) intraperitoneally thrice a week after the second immunisation. This process lasted for 38 days as indicated. MTX was used as a positive control, which was based on a recent study that reported MTX (35 mg/kg, twice per week, a total of 70 mg/kg per week) could significantly alleviate joint swelling after treatment for 3.5 weeks.^36^ The dosage of ATT we used in this study was based on our previous research and proved to be effective in suppressing tumour growth without toxic effects in vivo.^37^ Each paw of the mice was evaluated thrice a week during ATT treatment period. The specific scoring criteria of arthritis score are as follows referring to our previous study^38^ and reported article^39^: normal (0); detectable arthritis with erythema (1); evident swelling and redness (2); severe swelling and redness (3); maximum swelling as well as a deformity with ankylosis (4). Arthritis incidence was presented as the percentage of mice with arthritis within all the mice in each group.

### 2.2 Histological analysis

On the indicated day after treatment, experimental animals were sacrificed with euthanasia, and the paws of mice were photographed. Joints were gained and fixed with 4% paraformaldehyde for 24 h before decalcification for 1 month, embedded in paraffin and sectioned. Safranin O (SO) staining was used for measuring the cartilage area of mouse joints. Haematoxylin and eosin staining was performed and scored by the pathologists based on the reported scoring system,^40^ where four high‐power magnification fields and intra‐articular inflammatory cell areas were counted to determine the percentage of intra‐articular inflammatory cell area.

### 2.3 Synovial tissues and cell culture

In this study, primary RA‐FLSs were cultured from four RA patients’ synovial tissues as we described previously.^41^ An immortalised RA‐FLSs cell line, MH7A, was purchased from Jennio Biotech Co., Ltd. (China). Cells were cultured as we reported previously,^35^ and passages 3–7 of the primary cells were adopted for the subsequent studies.

### 2.4 Immunofluorescence assay

For identification of primary RA‐FLSs and related protein staining, cells were cultured in 96‐well culture plates (6000 cells per well) before stimulation with or without ATT (10 μM) for 24 h. Then, RA‐FLSs were fixed and incubated with 0.1% Triton X‐100 for 15 min before being blocked with 1.5% bovine serum albumin (Merck, Germany) for 45 min at room temperature (RT). RA‐FLSs were incubated overnight with rabbit anti‐vimentin antibody (ab128507, Abcam, UK), rabbit anti‐E‐cadherin (20874‐1‐AP, Proteintech, China) and rabbit anti‐N‐cadherin (22018‐1‐AP, Proteintech, China) antibodies and then further probed with Alexa Fluor 488‐labelled immunoglobulin G (IgG) antibody (A‐11008, Invitrogen, USA) before incubation with 4′,6‐diamidino‐2‐phenylindole (AAT Bioquest, USA) for 30 min at RT. The images of RA‐FLSs were captured by a fluorescence microscope.

### 2.5 Cell viability assay

Primary RA‐FLSs and MH7A were cultured in 96‐well culture plates (6000 cells per well) for 24 h before incubation with different dosages (0, 2.5, 5, 7.5, 10 and 15 μM) of ATT for 24 h. Then, 10 μl of Cell Counting Kit‐8 (CCK‐8) solution (APExBIO Corporation, USA) was added and co‐cultured for 1.5 h. Absorbance was tested by Synergy H1 Multimode Reader (BioTek, USA) at 450 nm.

### 2.6 5‐ethynyl‐2'‐deoxyuridine (EdU) assay

RA‐FLSs were cultured in 96‐well culture plates (6000 cells per well) for 24 h. Cells were stimulated with various dosages (0, 5, 10 and 15 μM) of ATT for 24 h. After treatment, EdU‐positive cells were measured by EdU Staining Proliferation Kit purchased from APExBIO Corporation. Fluorescence images were captured by a fluorescence microscope.

### 2.7 Cell cycle and apoptosis analysis

RA‐FLSs were cultured in 6‐well culture plates (2.0 × 10^5^ cells per well), and cells were stimulated with ATT for 24 h. Then, RA‐FLSs were collected and stained by a cell cycle detection kit and an apoptosis detection kit (MultiSciences, China). The cell cycle ratio and apoptotic cells of RA‐FLSs were determined by CytoFLEX LX.

### 2.8 Migration and invasion assays

These assays were carried out as we reported previously.^42^ In short, the chamber was precoated with or without Matrigel for 4 h. A total of 3.0 × 10^4^ RA‐FLSs in Dulbecco's modified Eagle medium were added to the upper chamber after ATT treatment for 24 h before complete medium was added to the lower chamber. After 24 and 48 h co‐culture for migration assay and invasion assay, respectively, migrated and invaded RA‐FLSs were fixed for 30 min, and then stained with crystal violet for 30 min. The stained RA‐FLSs were photographed by microscope and counted by ImageJ software.

### 2.9 Quantitative real‐time PCR (RT‐qPCR) assay

RA‐FLSs were dealt with as cell cycle and apoptosis analysis described above. Total RNA was isolated before reversely transcribed to cDNA, and then RT‐qPCR was performed as we reported.^43^ The sequences of primer used in RT‐qPCR are presented in Table S1.

### 2.10 Western blot analysis

Cells were dealt with as cell cycle and apoptosis analysis described above. Whole‐cell extracts were prepared, and then separated by sodium dodecyl sulfate‐polyacrylamide gel electrophoresis (SDS‐PAGE) before being transferred to nitrocellulose membranes (Millipore, USA). Antibodies used in this study included cyclin‐dependent kinase 1 and 2 (CDK1 and CDK2; DF6024 and AF6237, Affinity, USA), cyclinA2 (18202‐AP, Proteintech, China), cyclinB1 (28603‐1‐AP, Proteintech, China), B‐cell lymphoma‐2 (Bcl‐2; 12789‐1‐AP, Proteintech, China), Bcl‐2 associated X (Bax; 50599‐2‐Ig, Proteintech, China), vimentin (ab128507, Abcam, UK), E‐cadherin (20874‐1‐AP, Proteintech, China), N‐cadherin (22018‐1‐AP, Proteintech, China), ICAM2 (DF6772, Affinity, USA), PI3K (AF6242, Affinity, USA), p‐PI3K (AF3242, Affinity, USA), AKT (4691, CST, USA), p‐AKT (4060, CST, USA), p300 (AF5360, Affinity, USA), METTL3 (ab195352, Abcam, UK) and β‐actin (4970, CST, USA), as well as anti‐rabbit IgG horseradish peroxidase‐linked antibody (7074, CST, USA). ChemiDoc XRS+ (Bio‐Rad, USA) was adopted for capturing images.

### 2.11 RNA‐sequencing (RNA‐seq) analysis

RA‐FLSs were dealt with as cell cycle and apoptosis analysis described above. After treatment, cells were harvested, and RNA‐seq was performed and analysed as we reported previously.^35^

### 2.12 Bioinformatics analysis

mRNA expression profiles of RA synovial fibroblasts (RASF) and healthy synovial fibroblasts (HSF) were obtained from GSE21959 and GSE29746 in the GEO database (http://www.ncbi.nlm.nih.gov/geo/). Then, the mRNA expression data were transformed and normalised for further analyses. The packages in R were used for gene set enrichment analysis (GSEA). Here, statistical significance was calculated as *p*‐value < .05. The bar plot, bubble plot and chord were generated using R software to view the top terms. The relations between gene expressions were measured by Spearman's rank correlation tests.

### 2.13 Cell infection and transfection

ICAM2 overexpression (OE‐ICAM2) lentivirus, plasmids containing shRNA targeting ICAM2, METTL3 and p300, as well as overexpression of METTL3 were obtained from GeneChem Corporation (China), and the sequences are listed in Table S2. Primary RA‐FLSs were cultured in 6‐well culture plates (3.0 × 10^5^ cells per well). For cell infection, RA‐FLSs were infected with OE‐ICAM2 lentivirus to establish a stable overexpressed ICAM2 cell line as we previously described.^34^ For cell transfection, 1 μg of targeted plasmids were transfected into RA‐FLSs with Lipofectamine 3000 (Invitrogen, USA). The mRNA and protein expression of ICAM2, METTL3 and p300 were determined by RT‐qPCR and Western blotting.

### 2.14 Methylated RNA immunoprecipitation assay

RA‐FLSs were cultured in ten 10 cm cell culture dishes and reached a density of 80% before stimulation with or without ATT for 24 h. Then, cells were harvested, and methylated RNA immunoprecipitation (MeRIP) was performed using riboMeRIP m6A Transcriptome Profiling Kit (RIBOBIO, China) according to the manufacturer's instructions. Briefly, total RNAs were extracted and fragmented into approximately 200 nucleotides. Then, the fragments were immunoprecipitated with m6A antibody‐conjugated magnetic beads. The collected RNAs from the input, IgG and immunoprecipitation (IP) samples were used for further analysis by RT‐PCR and RT‐qPCR. The sequences of primers employed in RT‐PCR and RT‐qPCR are presented in Table S1.

### 2.15 Chromatin immunoprecipitation (ChIP) assay

RA‐FLSs were cultured in ten 10 cm cell culture dishes and reached a density of 80% before being stimulated with or without ATT for 24 h. Then, cells were harvested, and ChIP assay was performed using SimpleChIP^®^Plus Enzymatic ChIP Kit (9005S, CST, USA) according to the manufacturer's instructions. Briefly, chromatin was crosslinked and extracted from RA‐FLSs. Subsequently, the extracted chromatin was used for fragmentation and immunoprecipitation with p300 (54062, CST, USA) antibody‐conjugated magnetic beads. The eluted DNA was purified and used for further analysis by RT‐PCR and RT‐qPCR. The primers for PCR and qPCR are shown in Table S1.

### 2.16 Statistical analysis

The data in the current study are shown as the mean ± standard deviation, and statistics were executed as we previously reported.^34^ All data in this study are from at least three independent experiments, and *p* < .05 was considered statistically significant.

## 3 RESULTS

### 3.1 ATT significantly relieves CIA in mice

**FIGURE 1:**
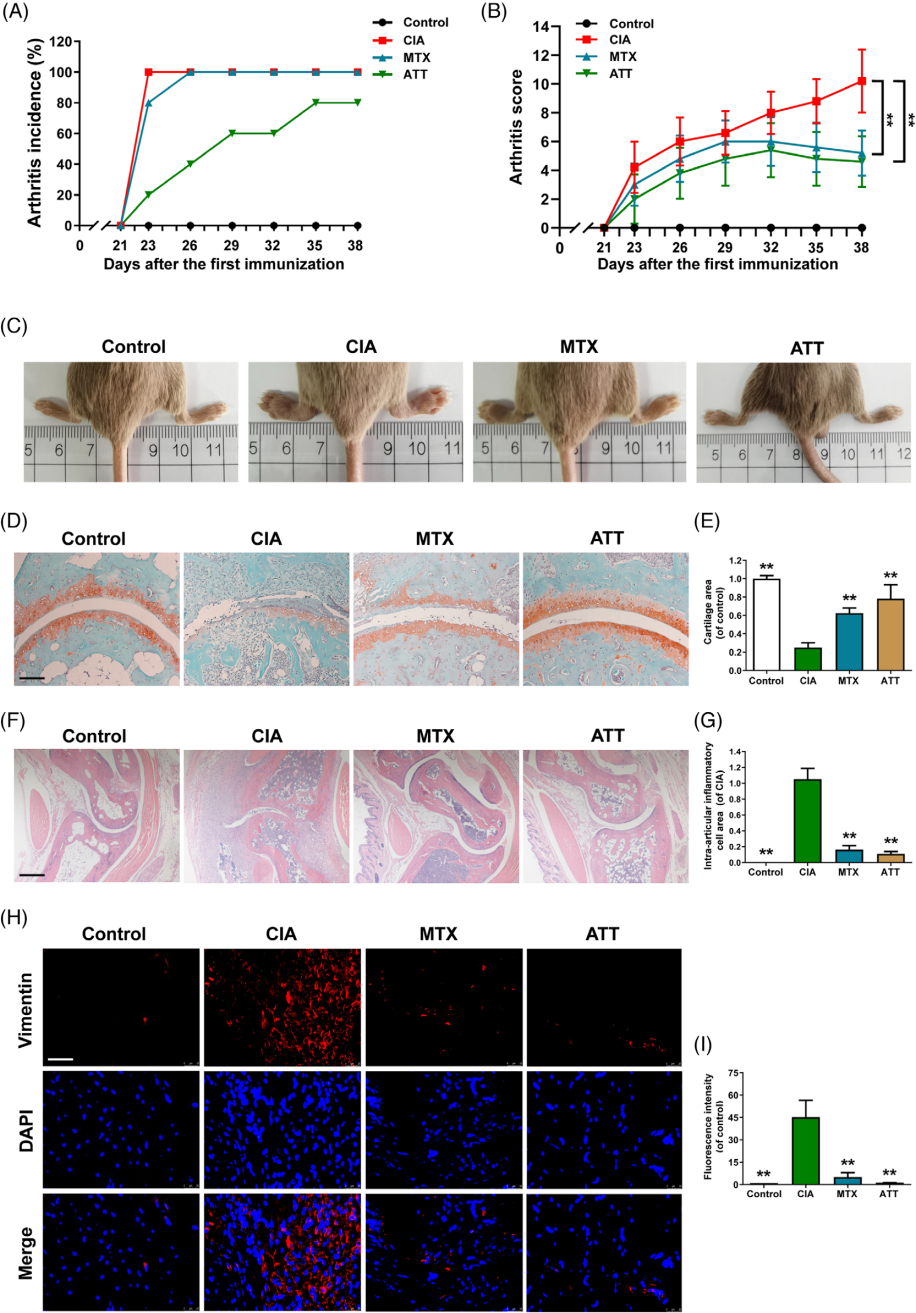
Artemisitene (ATT) significantly relieves collagen‐induced arthritis (CIA) in mice. Mouse models with CIA were established, and methotrexate (MTX) or ATT treatment was carried out three times per week after the second immunisation for the indicated duration. (A and B) The effect of ATT treatment on the incidence (A) and paw severity (B) of CIA mice were assessed after the second immunization (*n* = 5). (C–I) The swollen joints (C), safranin O (SO) staining (D and E), haematoxylin and eosin (H&E) staining (F), intra‐articular inflammatory cell area (G), and immunofluorescence of vimentin (H and I) of paw sections are shown after ATT treatment for 17 days. Scale bar of SO staining, H&E staining and immunofluorescence are 25, 125 and 12.5 μm, respectively. ^*^ *p* < .05 and ^**^ *p* < .01 versus CIA group

The CIA mouse model has the most similar clinical manifestations and laboratory parameters to the RA patients and is widely used for RA studies.^44^ Therefore, CIA mice were employed in the current study to detect the anti‐RA effectiveness of ATT in vivo. Our results showed that all of the mice in the CIA and MTX groups developed arthritis on days 23 and 26 after the first immunization, respectively, while the mice in the ATT group did not develop RA during the experimental period (Figure 1A). We also measured the body weight of mice after ATT treatment, and the data showed no significant differences between the CIA group and the ATT group (Figure S1A). In addition, arthritis scores and joint swelling from the MTX group and ATT group were decreased and mitigated, respectively, compared with the CIA group (Figure 1B,C). Moreover, SO staining showed that cartilage damage in mouse joints from the MTX and ATT groups was alleviated compared with that in the CIA group (Figure 1D,E). Furthermore, we also revealed that the synovial inflammatory infiltration and cartilage erosion in the MTX group and ATT group were alleviated compared with those in the CIA group (Figure 1F,G). We further showed that vimentin, a marker of FLSs in synovial tissue from the ATT group was remarkably reduced after ATT treatment (Figure 1H,I), indicating that ATT could inhibit the hyperplasia of abnormal FLSs in synovial tissue. These discoveries demonstrated that ATT might be a novel anti‐RA drug candidate.

### 3.2 ATT inhibits proliferation and induces apoptosis of RA‐FLSs

**FIGURE 2:**
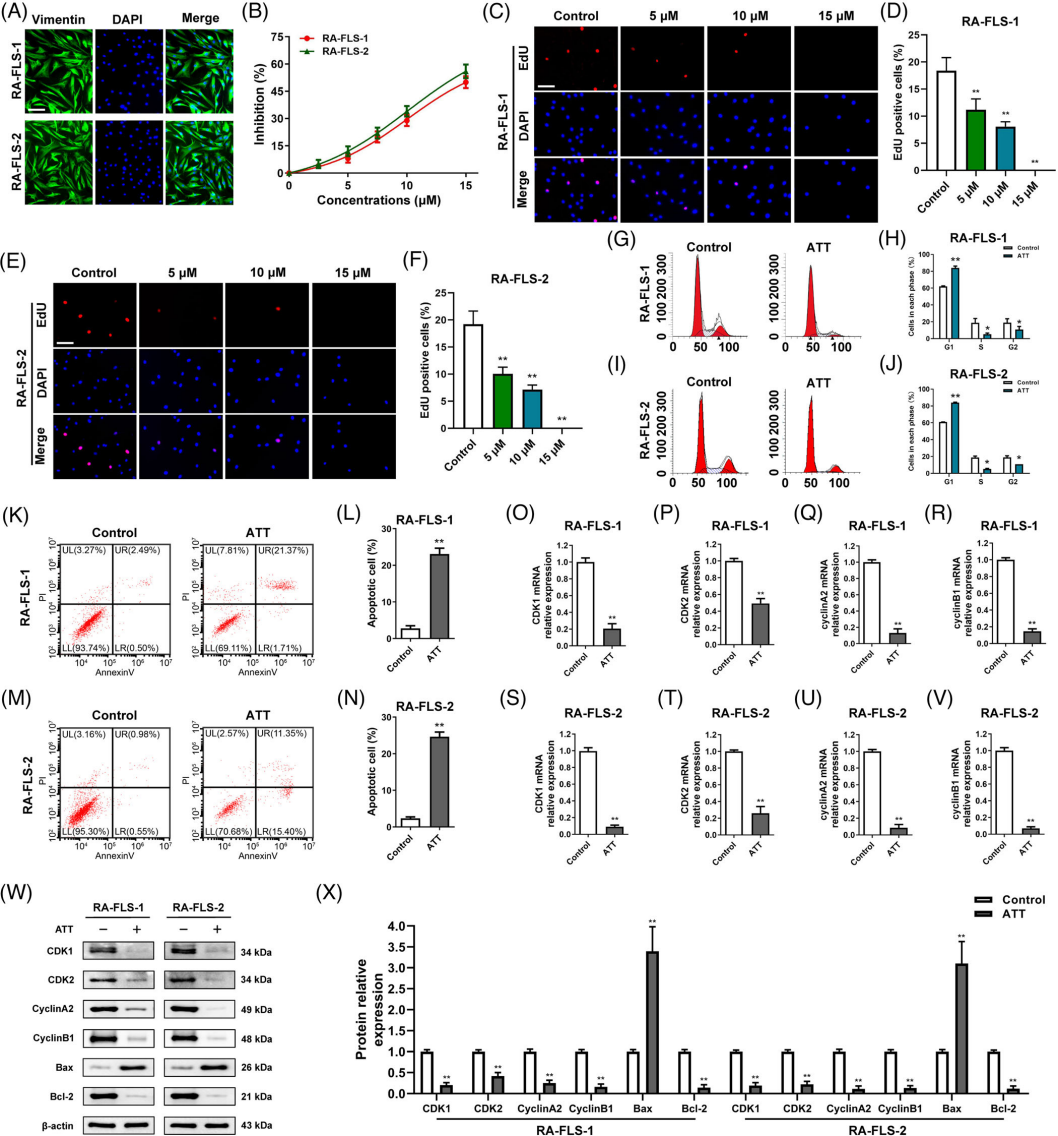
Artemisitene (ATT) inhibits proliferation and induces apoptosis of rheumatoid arthritis‐fibroblast‐like synoviocytes (RA‐FLSs). (A) Synovial tissues were obtained from two patients with RA, and RA‐FLSs were identified by immunofluorescence assay using monoclonal antibody vimentin. Scale bar: 25 μm. (B) RA‐FLSs were stimulated with multiple concentrations (2.5, 5, 7.5, 10 and 15 μM) of ATT for 24 h, then CCK‐8 assay was carried out to measure the cell viability (*n* = 5). (C–F) RA‐FLSs were stimulated with 5, 10 and 15 μM of ATT for 24 h. Representative photographs of the EdU‐positive cells are shown, and the statistical differences were measured after ATT stimulation using EdU assay (*n* = 3). Scale bar: 25 μm. (G–N) RA‐FLSs from two RA patients were stimulated with ATT for 24 h. Flow cytometry was adopted to estimate the cell cycle (G–J) and apoptotic cells (K–N) after ATT treatment (*n* = 3). (O–V) The relative mRNA expression of genes associated with cell cycle after ATT treatment for 24 h in RA‐FLSs was measured by RT‐qPCR (*n* = 3). (W and X) The relative protein expression of proteins associated with cell cycle and apoptosis after ATT treatment for 24 h in RA‐FLSs was measured by Western blot (*n* = 3). ^*^ *p* < .05 and ^**^ *p* < .01 versus control

Inspired by the results obtained in vivo, we next investigated the anti‐RA effect of ATT in vitro. Firstly, we obtained synovial tissues from four patients with RA and cultured primary RA‐FLSs for further study (Figures 2A and S2A). We found that ATT significantly suppressed the viability of RA‐FLSs from four patients (namely, RA‐FLS‐1, RA‐FLS‐2, RA‐FLS‐3 and RA‐FLS‐4) and an immortalised RA‐FLSs MH7A in a dosage‐dependent manner (Figures 2B and S2B). However, MTX, a clinical drug used for RA treatment, had no obvious inhibitory effect on RA‐FLSs viability (Figure S3A,B) and MH7A cells (Figure S3C) under the same conditions. In addition, we revealed that ATT treatment led to the inhibition of DNA replication in RA‐FLSs (Figures 2C–F and S4A,B). Besides, we also found that ATT stimulation promotes cell cycle arrest (Figure 2G–J) and apoptosis (Figures 2K–N and S5A,B) in detected RA‐FLSs. Interestingly, genes related to the cell cycle, including CDK1, CDK2, cyclinA2 and cyclinB1, in RA‐FLSs were also decreased after ATT stimulation (Figure 2O–V). Moreover, protein expression analysis also confirmed that proteins related to the cell cycle were all downregulated after ATT stimulation (Figure 2W,X). Furthermore, we also found that the anti‐apoptotic protein Bcl‐2 level was reduced, while the pro‐apoptotic protein Bax level was increased in RA‐FLSs after ATT stimulation (Figure 2W,X), supporting the results of apoptosis analysis. These results verified that ATT suppresses proliferation and induces apoptosis in RA‐FLSs.

### 3.3 ATT inhibits migration and invasion by suppressing epithelial–mesenchymal transition (EMT) in RA‐FLSs

**FIGURE 3:**
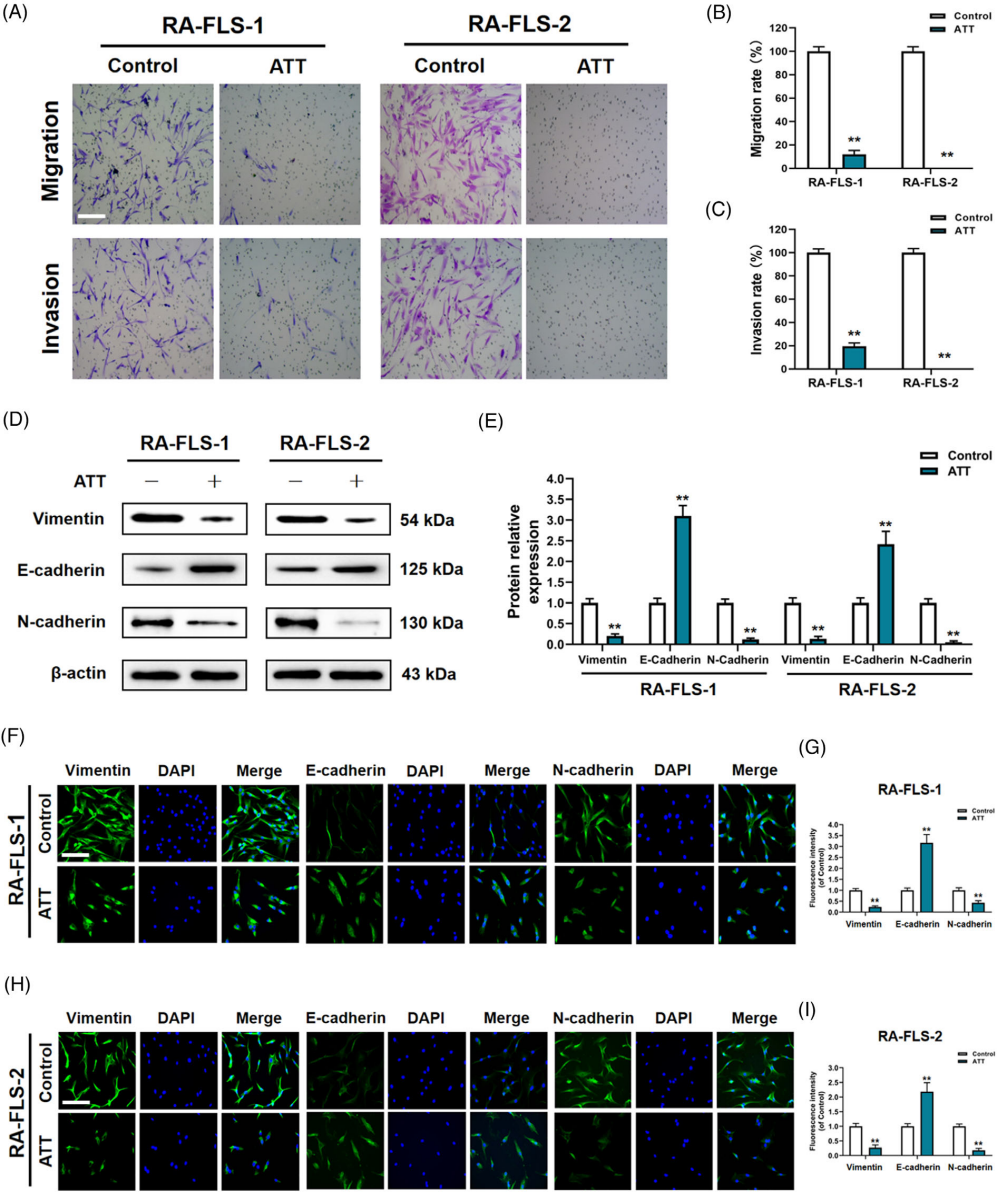
Artemisitene (ATT) inhibits migration and invasion by suppressing epithelial–mesenchymal transition (EMT) in rheumatoid arthritis‐fibroblast‐like synoviocytes (RA‐FLSs). RA‐FLSs from two patients with RA were treated with ATT (10 μM) for 24 h. (A–C) Representative photographs of migrant and invasive cells (A) are shown, and the statistical differences were measured after ATT stimulation (B and C) (*n* = 3). Scale bar: 50 μm. (D and E) The relative protein levels of vimentin, E‐cadherin and N‐cadherin were measured by Western blot (*n* = 3). (F–I) The representative photographs of vimentin, E‐cadherin and N‐cadherin expression were measured by immunofluorescence assay. Scale bar: 50 μm. ^**^ *p* < .01 versus control

RA‐FLSs have the tumour‐like biological characteristics of abnormal migration and invasion. To study the effectiveness of ATT on migration and invasion of RA‐FLSs, we demonstrated that ATT treatment results in significant suppression of migration and invasion in RA‐FLSs (Figures 3A–C, S6A–C and S7A,B), suggesting that ATT has strong efficiency to inhibit RA‐FLSs migration and invasion. It is known that migration of activated RA‐FLSs undergoing tumour‐like EMT process is considered a reason for spreading arthritis destruction to distant joints.^45^, ^46^ Therefore, we also tested the related markers of EMT, including vimentin, E‐cadherin and N‐cadherin, and demonstrated that ATT reduces vimentin and N‐cadherin levels, while enhancing E‐cadherin level in RA‐FLSs (Figure 3D,E). We further observed vimentin, E‐cadherin and N‐cadherin expression in RA‐FLSs after ATT treatment by immunofluorescence staining under the same conditions and gained similar Western blot results (Figure 3F–I). These findings revealed that ATT has the capacity to inhibit migration and invasion in RA‐FLSs by inhibiting the activation of EMT progression.

### 3.4 RNA‐seq and bioinformatics analysis recognised the ATT‐regulated genes associated with RA development in FLSs

**FIGURE 4:**
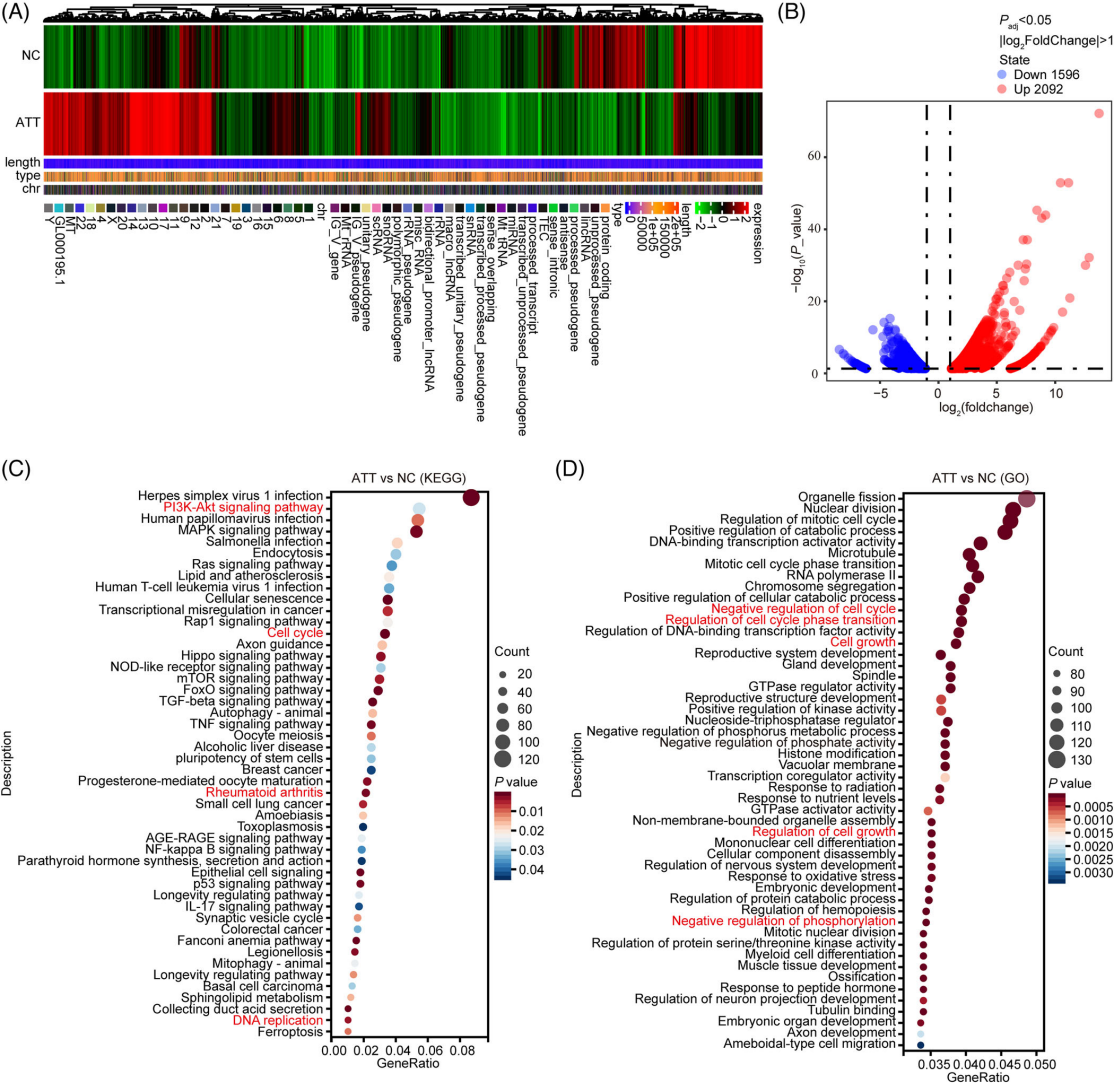
RNA‐sequencing (RNA‐seq) data analysis of artemisitene (ATT)‐treated rheumatoid arthritis‐fibroblast‐like synoviocytes (RA‐FLSs). RA‐FLSs were treated with ATT (10 μM) for 24 h, and non‐treated RA‐FLSs were used as normal control. Cells were harvested for mRNA sequencing analysis after treatment. (A) The heatmap shows the differentially expressed gene information between the ATT‐treated group and the control group. (B) Volcano diagram shows the differential expression of genes between ATT‐treated group and control group. (C) Kyoto Encyclopedia of Genes and Genomes (KEGG) enrichment analysis showed the differentially enriched signalling pathways, including PI3K–AKT, RA and DNA replication pathways. (D) Gene Ontology (GO) enrichment analysis showed the differential genes were enriched in biological processes, molecular functions and cellular components, including regulation of cell cycle, growth and phosphorylation

**FIGURE 5:**
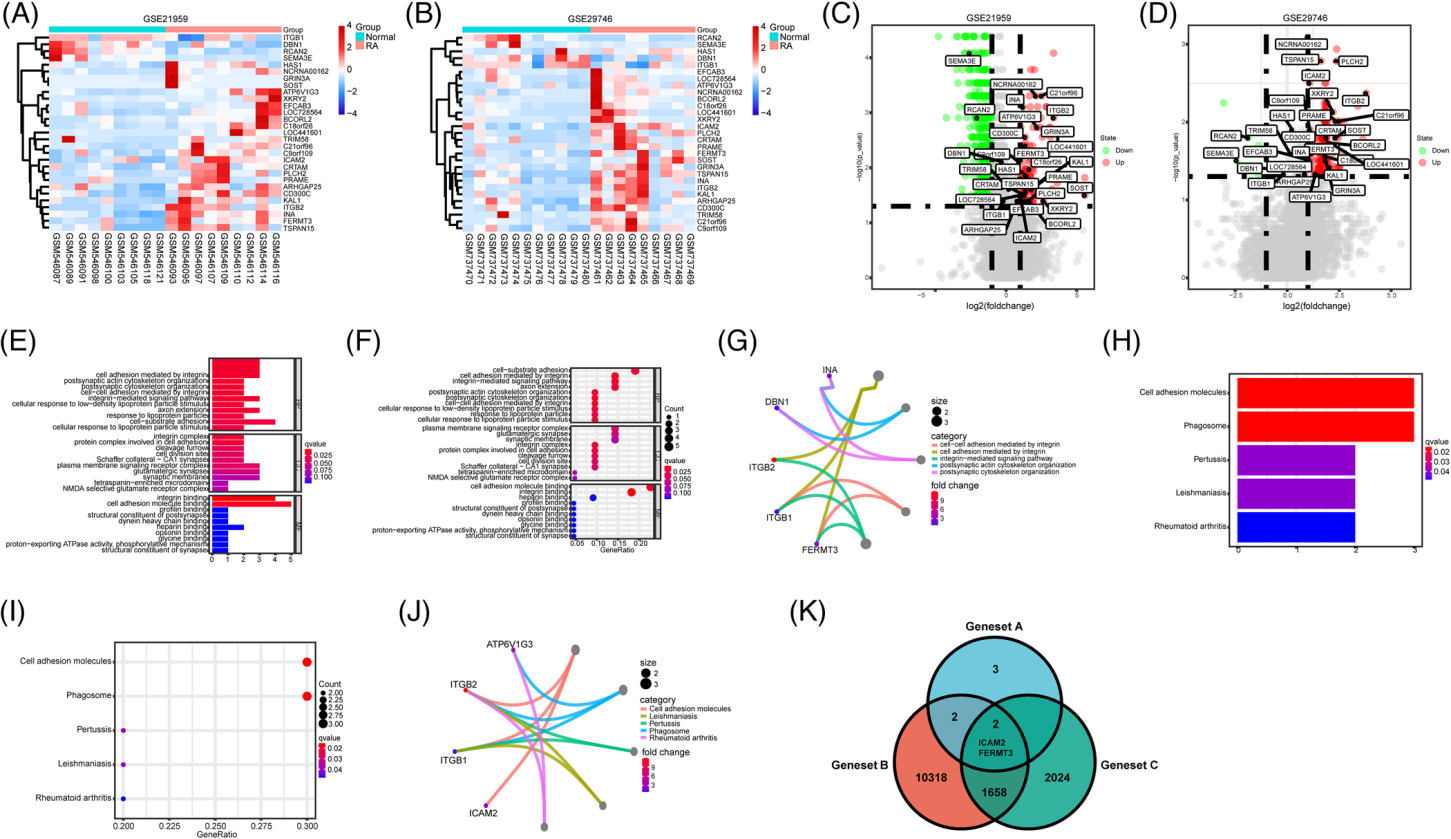
Bioinformatics analysis recognised the rheumatoid arthritis (RA) development associated genes in fibroblast‐like synoviocytes (FLSs). (A and B) The expression patterns of 29 differentially expressed genes between RA synovial fibroblasts (RASF) and healthy synovial fibroblasts (HSF) in GSE21959 and GSE29746, respectively. (C and D) Volcano plots of differentially expressed genes between RASF and HSF in GSE21959 and GSE29746, respectively. (E–J) Bar plots, bubble plots and chords show the top terms of the enriched Gene Ontology (GO) sets and Kyoto Encyclopedia of Genes and Genomes (KEGG) pathways. (K) The intersection of genes in artemisitene (ATT)‐treated RA‐FLSs (Geneset A) identified in gene set enrichment analysis (GSEA) (Geneset B) and genes correlated with clinical characteristics and treatment response of RA patients (Geneset C)

To identify the differentially expressed genes (DEGs) of ATT in suppressing RA‐FLSs, RNA‐seq data analysis of ATT‐treated RA‐FLSs was performed. The differential analysis revealed the DEGs with expression levels, length, type and chromosomal position after ATT stimulation (Figure 4A), and found a total of 1596 downregulated genes and 2092 upregulated genes (fold change ≥ 2, *p* < .05) (Geneset A) (Figure 4B). Besides, Kyoto Encyclopedia of Genes and Genomes enrichment analysis revealed the differentially enriched signalling pathways, such as PI3K–AKT, RA and DNA replication pathways (Figure 4C). Gene Ontology enrichment analysis revealed the differential genes were enriched in biological processes, molecular functions and cellular components, such as regulation of cell cycle, growth and phosphorylation (Figure 4D). In addition, the differential analyses were conducted according to the RNA‐seq data obtained from two GEO datasets (GSE21959 and GSE29746). A total of 2355 and 200 DEGs were screened between RASF and HSF in GSE21959 and GSE29746, respectively. There are 29 common genes of DEGs identified from GSE21959 and GSE29746 in RASF relative to HSF, among which 25 genes are upregulated genes and four genes are downregulated (Figure 5A–D). Then, GSEA were performed and suggested that the 29 genes concerning the regulation of cell adhesion molecules were related to biological processes and functions, and participated in the modulation of RA. Moreover, FERMT3, ITGB1, ITGB2, DBN1, INA, ICAM2 and ATP6V1G3 (Geneset B) were identified as key genes in RA development (Figure 5E–J). Subsequently, we found 11 980 genes (Geneset C) that were related to clinical characteristics and treatment responses of patients with RA according to a previously reported database (https://peac.hpc.qmul.ac.uk/). There are two common genes, ICAM2 and FERMT3, at the intersection of these three gene sets (Figure 5K), suggesting that these genes might participate in the biological process of RA‐FLSs.

### 3.5 ATT attenuates the ICAM2/PI3K/AKT/p300 pathway in RA‐FLSs

**FIGURE 6:**
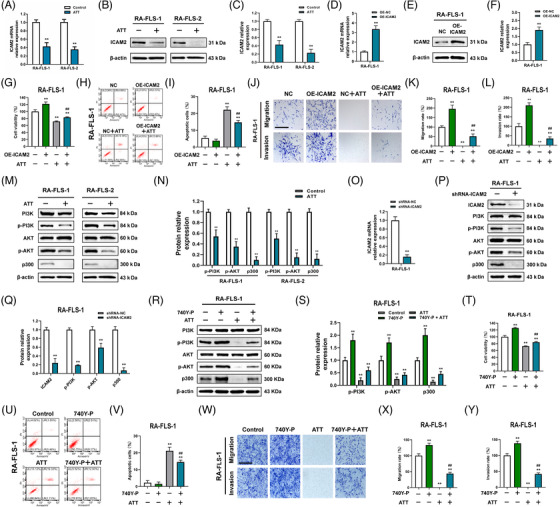
Artemisitene (ATT) attenuates intercellular adhesion molecule 2 (ICAM2)/PI3K/AKT/p300 pathway in rheumatoid arthritis‐fibroblast‐like synoviocytes (RA‐FLSs). RA‐FLSs from two patients with RA were stimulated with ATT (10 μM) for 24 h. (A–C) The relative mRNA (A) and protein (B and C) expression of ICAM2 after ATT treatment was measured by RT‐qPCR and Western blot in RA‐FLSs (*n* = 3). (D–F) RT‐qPCR (D) and Western blot (E and F) showing the overexpression efficiency of ICAM2 in RA‐FLSs. (G) RA‐FLSs with or without ICAM2 overexpression (OE‐ICAM2) were stimulated with ATT, then CCK‐8 assay was carried out to measure the cell viability (*n* = 5). (H–L) Representative photographs showing the apoptotic (H and I), migrant and invasive cells (J–L) after ATT stimulation in RA‐FLSs with or without OE‐ICAM2 (*n* = 3). Scale bar: 40 μm. (M and N) RA‐FLSs from two patients with RA were stimulated with ATT (10 μM) for 24 h. Western blot showing the relative levels of PI3K, p‐PI3K, AKT, p‐AKT and p300. (O–Q) RT‐qPCR (O) and Western blot (P and Q) showing the knockdown efficiency of ICAM2 in RA‐FLSs, and the relative levels of PI3K, p‐PI3K, AKT, p‐AKT and p300 after knockdown of ICAM2 in RA‐FLSs were measured by Western blot (P and Q) (*n* = 3). (R and S) The relative levels of PI3K, p‐PI3K, AKT, p‐AKT and p300 after ATT treatment with or without 740Y‐P in RA‐FLSs were measured by Western blot (*n* = 3). (T) RA‐FLSs with or without 740Y‐P were stimulated with ATT, then CCK‐8 assay was carried out to measure the cell viability (*n* = 5). (U–Y) Representative photographs showing the apoptotic (U and V), migrant and invasive cells (W–Y) after ATT stimulation in RA‐FLSs with or without 740Y‐P treatment (*n* = 3). Scale bar: 40 μm. ^**^ *p* < .01 versus control or negative control (NC); ^##^ *p* < .01 versus NC + ATT or ATT

Based on the RNA‐seq and bioinformatics analysis data, we next detected the effect of ATT on ICAM2 and FERMT3 mRNA expression. Interestingly, ATT conspicuously decreases the expression of ICAM2 in tested RA‐FLSs (Figure 6A–C), while increasing the mRNA expression of FERMT3 (Figure S8A), among which the differential expression of I is more significant. Therefore, we focused on investigating the role of ICAM2 in the potential mechanism of ATT management in RA‐FLSs. We next stably overexpressed ICAM2 in RA‐FLSs with lentivirus infection (Figure 6D–F). Interestingly, we found that overexpressed ICAM2 could alleviate the inhibitory impact of ATT on the proliferation and the apoptosis‐inducing effect of ATT in RA‐FLSs (Figure 6G,I). In addition, we further carried out migration and invasion assays under the same conditions and revealed similar results (Figure 6J–L). Overexpressed ICAM2 partly reverses the inhibitory impact of ATT on the proliferation, migration and invasion, and the apoptosis‐inducing effect of ATT in RA‐FLSs suggesting that an ICAM2‐independent mechanism may exist for the regulation of abnormal functions in RA‐FLSs by ATT. All the results indicated that ICAM2 is an important target of ATT in suppressing proliferation, migration and invasion, in addition to inducing apoptosis of RA‐FLSs.

Accumulating evidence shows that activation of PI3K/AKT signalling upregulates p300 expression^47^ and participates in modulating RA‐FLS migration and invasion.^48^, ^49^, ^50^ Moreover, p300 is involved in regulating the physiological function of fibroblasts, such as facilitating cell proliferation, migration and invasion.^51^ Therefore, we subsequently tested the activity of the PI3K/AKT/p300 axis after ATT stimulation. We found that ATT reduced p‐PI3K, p‐AKT and p300 levels in detected RA‐FLSs (Figure 6M,N), indicating that ATT suppressed the activation of PI3K/AKT/p300 pathway in RA‐FLSs. To further confirm whether ICAM2 affected the PI3K/AKT/p300 pathway, we knocked down ICAM2 in RA‐FLSs and found that p‐PI3K, p‐AKT and p300 levels were downregulated in ICAM2‐deficient RA‐FLSs (Figure 6O–Q). Furthermore, we also confirmed that activation of PI3K by 740Y‐P alleviated the inhibitory effect of ATT on the proliferation, migration and invasion, and apoptosis‐inducing effect of ATT in RA‐FLSs (Figure 6R–Y), suggesting that PI3K/AKT/p300 signalling is essential for ATT‐induced inhibition of abnormal functions in RA‐FLSs. In conclusion, we revealed that ATT suppresses proliferation, migration and invasion, and induces apoptosis in RA‐FLSs by regulating ICAM2/PI3K/AKT/p300 pathway.

### 3.6 ATT inhibits METTL3‐mediated m6A methylation of *ICAM2* mRNA and modulates METTL3/ICAM2/PI3K/AKT/p300 feedback loop in RA‐FLSs

**FIGURE 7:**
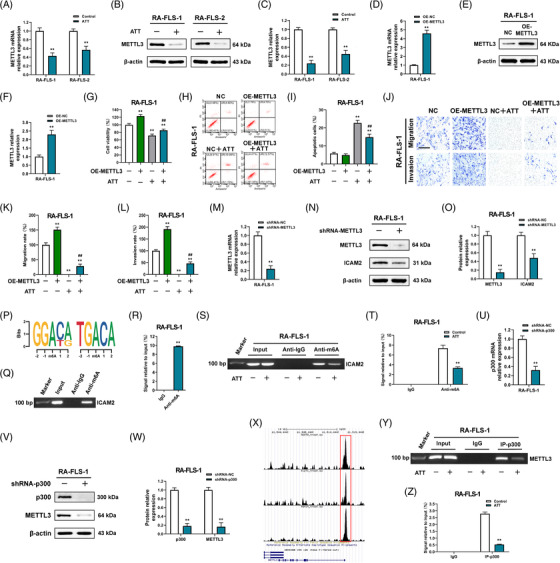
Artemisitene (ATT) inhibits METTL3‐mediated m6A methylation of intercellular adhesion molecule 2 (*ICAM2*) mRNA and modulates METTL3/ICAM2/PI3K/AKT/p300 feedback loop in rheumatoid arthritis‐fibroblast‐like synoviocytes (RA‐FLSs). (A–C) RA‐FLSs from two patients with RA were treated with ATT (10 μM) for 24 h. RT‐qPCR and Western blot showing the relative mRNA (A) and protein (B and C) expression of METTL3 after ATT treatment in RA‐FLSs (*n* = 3). (D–F) RT‐qPCR (D) and Western blot (E and F) showing the overexpression (OE) efficiency of METTL3 in RA‐FLSs. (G) RA‐FLSs with or without OE of METTL3 were stimulated with ATT for 24 h, then CCK‐8 assay was carried out to measure the cell viability (*n* = 5). (H–L) Representative photographs showing the apoptotic (H and I), migrant and invasive cells (J–L) after ATT stimulation in RA‐FLSs with or without METTL3 OE (*n* = 3). Scale bar: 40 μm. (M–O) RT‐qPCR (M) and Western blot (N and O) showing the knockdown efficiency of METTL3 in RA‐FLSs, and Western blot (N and O) showing the protein expression of ICAM2 after knockdown of METTL3 in RA‐FLSs (*n* = 3). (P) The m6A modification sites in the 3′‐untranslated region (UTR) of *ICAM2* mRNA were revealed through m6A‐REF‐seq and miCLIP in the RMVar and m6A‐Atlas databases. (Q and R) RNA immunoprecipitation (RIP) with anti‐m6A antibody showing the m6A modification of ICAM2 in RA‐FLSs by RT‐PCR (Q) and RT‐qPCR (R). (S and T) RIP with anti‐m6A antibody showing the m6A modification of ICAM2 in RA‐FLSs by RT‐PCR (S) and RT‐qPCR (T). (U–W) RT‐qPCR (U) and Western blot (V and W) showing the knockdown efficiency of p300 in RA‐FLSs (U–W), and Western blot (V and W) showing the protein expression of METTL3 after knockdown of p300 in RA‐FLSs (*n* = 3). (X) Chromatin immunoprecipitation sequencing (ChIP‐seq) data obtained from Cistrome Data Browser (http://cistrome.org/db/#/; CistromeDB: 36878, CistromeDB: 41271 and CistromeDB: 49414) showing that p300 could bind to the promoter regions of METTL3 in fibroblasts. (Y and Z) ChIP assay showing the inhibitory effect of ATT on METTL3 transcription directly regulated by p300 detected by RT‐PCR (Y) and RT‐qPCR (Z). ^**^ *p* < .01 versus control or negative control (NC); ^##^ *p* < .01 versus NC + ATT

m6A modification is one of the most important forms of internal modification to regulate RNA stability and translation efficiency.^12^ According to the data we obtained above, we wondered whether ICAM2 was regulated by m6A modification. According to the RNA‐seq data obtained from the GSE89408 dataset, we found that five m6A regulators, including IGF2BP3, YTHDF2, HNRNPC, METTL3 and RBM15, have the most relative relationship with ICAM2 in RA synovial tissues (Figure S9A). Moreover, we further confirmed the data using another single‐cell RNA‐seq data obtained from the GSE109449 dataset and found that the m6A writer METTL3 had the most positive correlation with ICAM2 in RA‐FLSs (Figure S9B). Therefore, we next detected METTL3 level after ATT treatment in RA‐FLSs. Intriguingly, we further revealed that ATT treatment decreased *METTL3* mRNA (Figure 7A) and protein expression (Figure 7B,C) in detected RA‐FLSs. Subsequently, to study the special role of METTL3 in the potential mechanism of ATT management in RA‐FLSs, we stably overexpressed METTL3 in RA‐FLSs (Figure 7D–F). Interestingly, we found that overexpressed METTL3 alleviated the inhibitory impact of ATT on proliferation, migration and invasion, in addition to the apoptosis‐inducing effect of ATT in RA‐FLSs (Figure 7G–L), implying that ATT might affect METTL3 to regulate ICAM2 expression in RA‐FLSs. To further investigate whether METTL3 affects ICAM2 expression, we silenced METTL3 expression in RA‐FLSs and indeed showed that ICAM2 was downregulated in METTL3‐deficient RA‐FLSs (Figure 7M–O). Furthermore, chr17:64002634(‐) and chr17:64002654(‐) were found to be m6A methylation sites in the 3′‐untranslated region (UTR) of *ICAM2* mRNA revealed through m6A‐REF‐seq and m6A individual‐nucleotide‐resolution cross‐linking and immunoprecipitation (miCLIP) in the RMVar (http://rmvar.renlab.org) and m6A‐Atlas (http://180.208.58.66/m6A‐Atlas/) databases (Figure 7P). Considering that the decrease in METTL3 expression induced by ATT might affect other mRNAs’ m6A sites, the m6A‐seq in RA‐FLSs was performed to reveal whether ATT treatment could specifically regulate the m6A methylation of *ICAM2* mRNA. By analysing the transcriptional region with the m6A peaks, the m6A methylated sites were mainly enriched in the 3′‐UTR (Figure S10A), which was consistent with a previous study on MH7A.^16^ Moreover, ATT could impair the m6A methylation located in the 3′‐UTR (Figure S10A). Then, a total of 2952 genes with 4408 differential m6A methylation peaks were identified after ATT treatment (Figure S10B). Interestingly, these genes were also shown to be involved in PI3K/AKT signalling (Figure S10C). Then, we compared the 2952 genes with differential m6A methylation after ATT treatment (Geneset A1), the 3684 DEGs after ATT stimulation (Geneset B1), the 11 980 genes correlated with RA in the PEAC database (https://peac.hpc.qmul.ac.uk/) (Geneset C1), and 5081 genes related to RA in Genecards database (https://www.genecards.org/) (Geneset D1). Interestingly, we obtained 85 genes from the intersection, and ICAM2 ranked first in the gene list (Figure S10D). In addition, the m6A‐seq showed an m6A methylation peak of *ICAM2* mRNA that started from chr17:64002623 to chr17:64002772 and covered the m6A methylation sites chr17:64002634(‐) and chr17:64002654(‐) revealed by bioinformatics analyses (Figure S10E). MeRIP together with RT‐qPCR and RT‐PCR confirmed m6A methylation of *ICAM2* mRNA (Figure 7Q,R), and further demonstrated that m6A methylation of *ICAM2* mRNA was impaired after ATT treatment in RA‐FLSs (Figure 7S,T). All these findings confirmed that ATT inhibits METTL3‐mediated m6A modification of *ICAM2* mRNA in RA‐FLSs.

**FIGURE 8:**
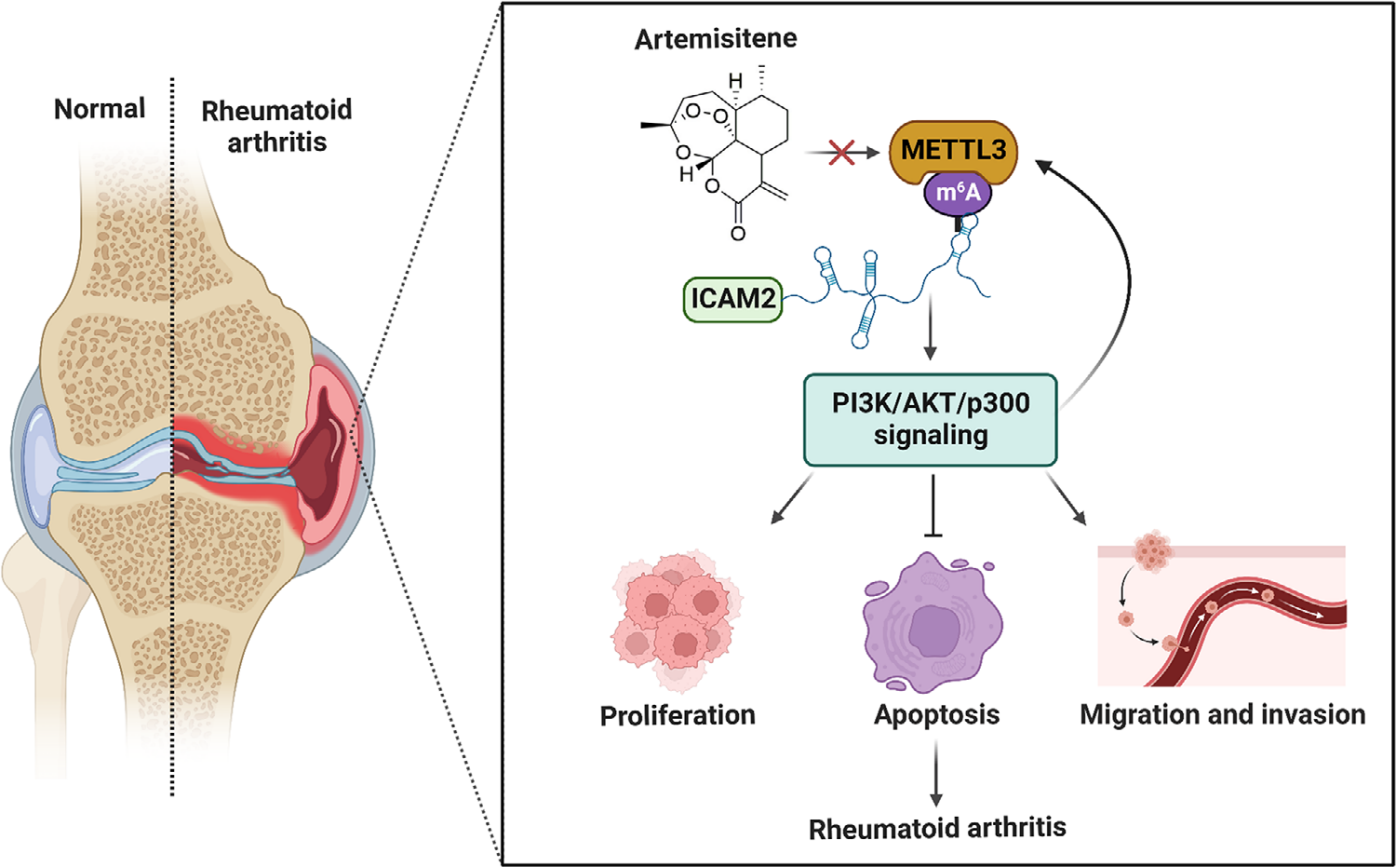
Underlying working model of artemisitene (ATT) in treating rheumatoid arthritis (RA). ATT regulates proliferation, apoptosis as well as migration and invasion of RA‐fibroblast‐like synoviocytes (RA‐FLSs) by modulating the METTL3/intercellular adhesion molecule 2 (ICAM2)/PI3K/AKT/p300 feedback loop, contributing to the suppression of RA progression

To further study the mechanism by which ATT modulates METTL3‐mediated m6A methylation of *ICAM2* mRNA in RA‐FLSs, we used the published RNA‐seq data gained from two GEO datasets (GSE29746 and GSE21959) to predict the transcription factors of METTL3. To our surprise, METTL3 has a strong positive correlation with p300 (Figure S11A,B), which is downstream of ICAM2/PI3K/AKT axis in RA‐FLSs, as determined above. In order to ascertain the relationship between METTL3 and p300, we silenced p300 in RA‐FLSs and showed that the knockdown of p300 led to a decrease in METTL3 levels (Figure 7U–W). According to the ChIP‐seq data gained from Cistrome Data Browser (http://cistrome.org/db/#/; CistromeDB: 36878, CistromeDB: 41271 and CistromeDB: 49414), we found that p300 could bind to the promoter regions of METTL3 in fibroblasts (Figure 7X), suggesting that p300 might directly promote METTL3 transcription. Interestingly, we further verified that p300 directly facilitates METTL3 transcription, which could be restrained by ATT treatment in RA‐FLSs by ChIP assay (Figure 7Y,Z). In addition, we also revealed that silencing ICAM2 and inhibiting the activation of PI3K in RA‐FLSs led to a decrease in METTL3 level (Figures S12A and S13A). Besides, we further confirmed that activation of PI3K by 740Y‐P significantly reversed the expression of METTL3 and ICAM2 reduced by ATT in RA‐FLSs (Figure S14A). These findings suggested the formation of METTL3/ICAM2/PI3K/AKT/p300c, which could be modulated by ATT (Figure S15A). Moreover, we found that ATT decreased the expression of ICAM2, METTL3 and p300 in synovial tissues of CIA mice (Figure S16A). Furthermore, bioinformatics analysis of RA clinical specimens in the PEAC RNA‐seq database revealed that METTL3, ICAM2 and p300 expression in RA synovial tissues was related to clinical characteristics and treatment responses, such as CRP, ESR and ΔCRP in RA patients (Figure S17A–C). These findings supported the role of target proteins regulated by ATT in RA progression, and provided strong evidence for the further clinical use of ATT. Taken together, we demonstrated that ATT modulates METTL3/ICAM2/PI3K/AKT/p300 feedback loop to suppress proliferation, migration and invasion, and induce apoptosis in RA‐FLSs (Figure 8).

## 4 DISCUSSION

Recently, early diagnosis and treatment, as well as alternative antirheumatic drugs have largely ameliorated RA management^52^ and long‐term prognosis.^53^ Nevertheless, prolonged therapy with antirheumatic drugs constantly results in resistance and side effects. Therefore, it is necessary to develop novel antirheumatic drugs for alleviating treatment failure in RA.^54^ In search of efficient drugs that can suppress RA with limited side effects on normal tissues, we discovered that the natural compound ATT exhibits potential for RA management due to its excellent inhibitory effect on cell proliferation, migration and invasion, as well as its apoptosis‐inducing effect in RA‐FLSs. Recently, a study showed that artesunate, another derivative of artemisinin, can inhibit RA‐FLSs migration and invasion,^27^ and its anti‐RA activity is inferior to that of ATT. Therefore, ATT would be a potential and safer drug candidate with the efficient activity of anti‐RA.

FLSs play a key role in pannus formation by migrating and invading cartilage and are the most critical factor in the pathologic process of RA. Abnormal proliferation, migration and invasion of FLSs are vital in RA pathogenesis.^1^ Here, we confirmed that ICAM2, selected as an RA development associated gene by bioinformatics analysis, was an important target of ATT and mediated the inhibitory effect of ATT in RA‐FLSs, suggesting that ATT might be an effective antirheumatic drug. The PI3K/AKT/p300 axis was reported to modulate cell proliferation^55^ and inflammatory responses.^47^, ^56^ In this work, we also observed the contribution of PI3K/AKT/p300 to modulating RA‐FLSs proliferation, migration and invasion, and apoptosis, further establishing the ICAM2/PI3K/AKT/p300 axis suppressed by ATT in RA‐FLSs.

To study the potential mechanism by which ATT suppresses ICAM2 expression, we revealed that METTL3 could activate the expression of ICAM2. It has been proven that METTL3 is essential for the progression of multiple cancers.^57^, ^58^, ^59^, ^60^, ^61^ Moreover, METTL3‐mediated m6A modification is crucial for the process of EMT and metastasis in gastric cancer^57^ and could be considered a promising therapeutic target to treat colorectal cancer.^60^ Nevertheless, the role and potential mechanism of METTL3 in RA are not fully understood. A recent study reported that the knockdown of METTL3 in RA‐FLSs leads to the cell cycle arrest and inhibition of migration and invasion,^15^ which supported our result that ATT inhibited METTL3 to regulate cell proliferation, apoptosis, migration and invasion of RA‐FLSs. We also clarified the stimulatory impact of METTL3 on ICAM2 level in RA‐FLSs and discovered the inhibitory effect of ATT on METTL3‐mediated m6A modification of *ICAM2* mRNA in RA‐FLSs, emphasising the regulated impact of m6A RNA methylation on the antirheumatic activity of ATT. Interestingly, p300 was demonstrated to be a transcription factor that could directly activate METTL3 transcription in neonatal rat cardiomyocytes and human gastric cancer,^52^, ^59^ which was consistent with our finding that the transcriptional coactivator p300 could enhance the expression of METTL3. These findings demonstrated that ATT suppresses the pathology of RA‐FLSs by modulating METTL3‐mediated m6A modification of *ICAM2* mRNA, thereby leading to inhibition of ICAM2/PI3K/AKT/p300 pathway. More importantly, we further verified that p300 directly facilitates METTL3 transcription, which could be restrained by ATT treatment in RA‐FLSs, forming the METTL3/ICAM2/PI3K/AKT/p300 positive feedback. Furthermore, this study also showed that METTL3, ICAM2 and p300 levels in RA synovium tissues were related to clinical characteristics and treatment response of RA patients, which supported the role of these target proteins regulated by ATT in RA progression and provided strong evidence for further clinical use of ATT. However, the unclear direct target of ATT is one of the limitations of this study, and we will further identify the direct target of ATT in RA‐FLSs in our future research. In addition, the effect of ATT on the immune system will also be investigated in our future study as artemisinin and its derivatives have been shown to have anti‐inflammatory and immunoregulatory properties.^62^

In summary, this study identifies that METTL3 mediates m6A methylation of *ICAM2* mRNA in RA‐FLSs, thereby activating PI3K/AKT/p300 signalling pathway and regulating RA progression. More importantly, we also confirmed that ATT has therapeutic potential for RA management by suppressing proliferation, migration and invasion, and inducing apoptosis in RA‐FLSs by modulating METTL3/ICAM2/PI3K/AKT/p300 feedback loop, supplying the foundation for the clinical application of ATT in RA therapy. Moreover, METTL3, ICAM2 and p300 might serve as biomarkers for the therapy response of RA patients.

## CONFLICT OF INTEREST

The authors declare they have no conflicts of interest.

## Supporting information

Supporting Information

## Data Availability

The data that support the findings of this study are openly available in the Supplementary Files. The datasets used and/or analyzed during the current study are available from the corresponding author on reasonable request.
