# Supplementary material for: Artemisitene suppresses rheumatoid arthritis progression via modulating METTL3‐mediated N6‐methyladenosine modification of ICAM2 mRNA in fibroblast‐like synoviocytes
Source: Clin Transl Med. 2022 Dec 19;12(12):e1148. doi: 10.1002/ctm2.1148 (PMC9763537; doi:10.1002/ctm2.1148)
Supplement: Supplementary file 1 — Supporting Information [file CTM2-12-e1148-s001.docx]

Supplementary material for

**Artemisitene suppresses rheumatoid arthritis progression via modulating METTL3-mediated N6-methyladenosine modification of *ICAM2* mRNA in fibroblast-like synoviocytes**

Jian Chen ^a, b, #, *^, Xian Lin ^a, b, #^, Juan He ^a, b^, Dandan Liu ^c^, Lianhua He ^a, b^, Miaomiao Zhang ^a, b^, Huijie Luan ^a, b^, Yiping Hu ^a, b^, Cheng Tao ^d, *^, Qingwen Wang ^a, b, *^

^a^ Department of Rheumatism and Immunology, Peking University Shenzhen Hospital, Shenzhen 518036, Guangdong, China

^b^ Shenzhen Key Laboratory of Inflammatory and Immunology Diseases, Shenzhen 518036, Guangdong, China

^c^ School of Basic Medical Science, Guangzhou University of Chinese Medicine, Guangzhou 510006, Guangdong, China

^d^ School of Pharmacy, Guangdong Medical University, Dongguan 523808, Guangdong, China

**^#^** These authors contributed equally to this work

* Corresponding author:

**Cheng Tao**: School of Pharmacy, Guangdong Medical University, No.1 Xincheng Blvd, Songshan Lake National High-tech Industrial Development Zone, Dongguan, Guangdong, 523808, China. Email: taocheng@gdmu.edu.cn

**Jian Chen** and **Qingwen Wang:** Department of Rheumatism and Immunology, Peking University Shenzhen Hospital, 1120 Lianhua Rd, Futian District, Shenzhen, Guangdong, 518036, China. Email: [chenjian@pkuszh.com](mailto:chenjian@jnu.edu.cn) (**Jian Chen**) and wqw_sw@163.com (**Qingwen Wang**); Phone: +86-0755-83923333

**This file includes:**

Table S1-2 and Figure S1-17.

| **Table S1** Primers of related genes | |
| --- | --- |
| **Genes** | **Primers (5′ - 3′)** |
| CDK1 | F: TGAGGTAGTAACACTCTGGTA |
|  | R: ATGCTAGGCTTCCTGGTT |
| CDK2 | F: CCTGGACACTGAGACTGA |
|  | R: CCGATGAGAATGGCAGAA |
| cyclin A2 | F: ACAGCCAGACATCACTAAC |
|  | R: GGAAGACAGGAACCTATCAA |
| cyclin B1 | F: TTGGTTGATACTGCCTCTC |
|  | R: TCTGACTGCTTGCTCTTC |
| ICAM2 | F: CCACAGCCACATTCAACA |
|  | R: AAGCAGAGCAGGACAGAT |
| METTL3 | F: CCCTATGGGACCCTGACAGA |
|  | R: CTGGTTGAAGCCTTGGGGAT |
| EP300 | F: ACCAGACTTGGCACCTTTC |
|  | R: AGGGCTTTGGTTCGGTAT |
| ICAM2-3’UTR | F: GACTGTGACAGGCAGCAGA |
|  | R: AAGTCCAGGTGTTTGTATTCG |
| METTL3-promoter | F: CGCTCTGCCAACACCTAA |
|  | R: GCTGAGCAAGACAGAAAAGTC |
|  |  |

| **Table S2** shRNA sequences of related genes | |
| --- | --- |
| **Genes** | **Primers (5′ - 3′)** |
| shRNA-ICAM2-1# | F: CCGGGAAACATTACTTGGTCTCAAACTCGAGTTTGAGACCAAGTAATGTTTCTTTTTG |
|  | R: AATTCAAAAAGAAACATTACTTGGTCTCAAACTCGAGTTTGAGACCAAGTAATGTTTC |
| shRNA-ICAM2-2# | F: CCGGGATGAGAAGGTATTCGAGGTACTCGAGTACCTCGAATACCTTCTCATC |
|  | R: AATTCAAAAAGATGAGAAGGTATTCGAGGTACTCGAGTACCTCGAATACCTTCTCATC |
| shRNA-ICAM2-3# | F: CCGGGAAGCAGGAGTCAATGAATTCCTCGAGGAATTCATTGACTCCTGCTTCTTTTTG |
|  | R: AATTCAAAAAGAAGCAGGAGTCAATGAATTCCTCGAGGAATTCATTGACTCCTGCTTC |
| shRNA-METTL3-1# | F: CCGGGCACTTGGATCTACGGAATCCCTCGAGGGATTCCGTAGATCCAAGTGCTTTTTG |
|  | R: AATTCAAAAAGCACTTGGATCTACGGAATCCCTCGAGGGATTCCGTAGATCCAAGTGC |
| shRNA-METTL3-2# | F: CCGGGCAAGAATTCTGTGACTATGGCTCGAGCCATAGTCACAGAATTCTTGCTTTTTG |
|  | R: AATTCAAAAAGCAAGAATTCTGTGACTATGGCTCGAGCCATAGTCACAGAATTCTTGC |
| shRNA-METTL3-3# | F: CCGGGCTCAACATACCCGTACTACACTCGAGTGTAGTACGGGTATGTTGAGCTTTTTG |
|  | R: AATTCAAAAAGCTCAACATACCCGTACTACACTCGAGTGTAGTACGGGTATGTTGAGC |
| shRNA-p300-1# | F: CCGGCCAGCCTCAAACTACAATAAACTCGAGTTTATTGTAGTTTGAGGCTGGTTTTTG |
|  | R: AATTCAAAAACCAGCCTCAAACTACAATAAACTCGAGTTTATTGTAGTTTGAGGCTGG |
| shRNA-p300-2# | F: CCGGGCCTTCACAATTCCGAGACATCTCGAGATGTCTCGGAATTGTGAAGGCTTTTTG |
|  | R: AATTCAAAAAGCCTTCACAATTCCGAGACATCTCGAGATGTCTCGGAATTGTGAAGGC |
| shRNA-p300-3# | F: CCGGCCCGGTGAACTCTCCTATAATCTCGAGATTATAGGAGAGTTCACCGGGTTTTTG |
|  | R: AATTCAAAAACCCGGTGAACTCTCCTATAATCTCGAGATTATAGGAGAGTTCACCGGG |


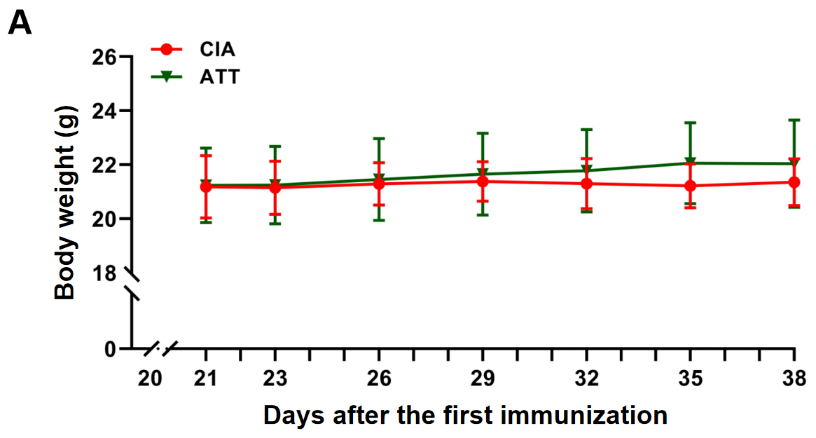


**Figure S1** Effect of ATT on the body weight of CIA mice. (A) Mouse models with CIA were established, and ATT treatment was carried out three times per week after the second immunization for the indicated duration. *n*=5.

**
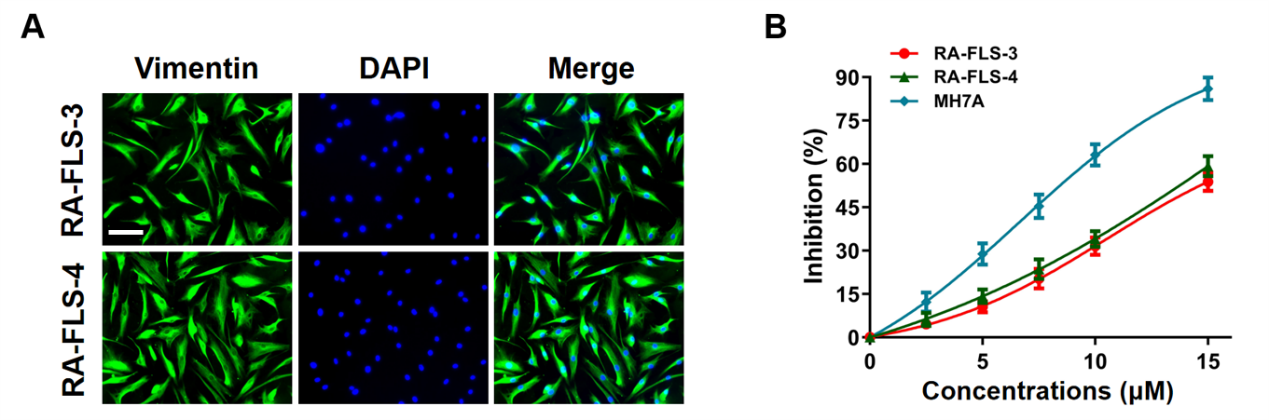
**

**Figure S2** Effect of ATT on the proliferation of RA-FLSs. (A) Synovial tissues were obtained from other 2 patients with RA, and RA-FLSs were identified by immunofluorescence assay using monoclonal antibody vimentin. (B) RA-FLS-3, RA-FLS-4, and MH7A cells were stimulated with multiple concentrations (2.5, 5, 7.5, 10 and 15 μM) of ATT for 24 h, then CCK-8 assay was carried out to determine the cell viability, *n*=5. Scale bar: 25 μm.

**
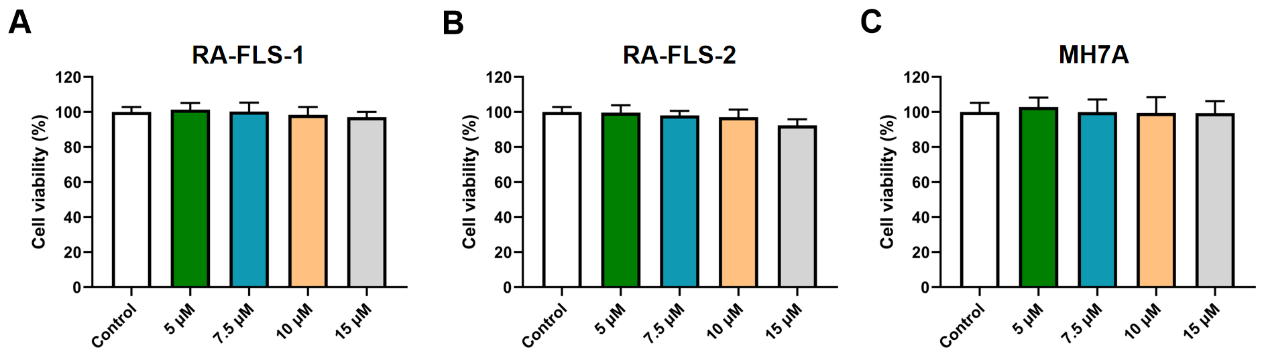
**

**Figure S3** Effect of MTX on the viability of RA-FLSs. (A, B) Primary RA-FLSs and MH7A were stimulated with multiple dosages (5, 7.5, 10 and 15 μM) of MTX for 24 h, then CCK-8 assay was carried out to determine the cell viability, *n*=5.

**
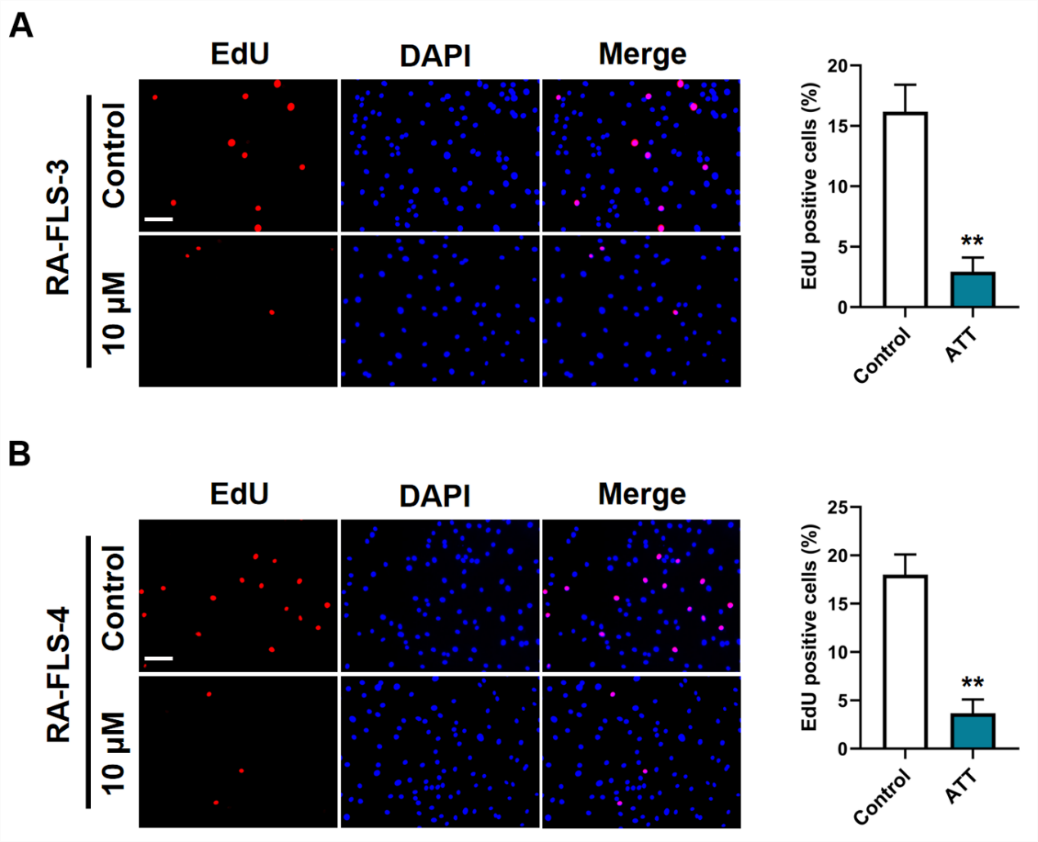
**

**Figure S4** ATT inhibits DNA replication of RA-FLSs. (A, B) Representative photographs of the EdU positive cells were shown, and the statistical differences were measured after 10 µM of ATT stimulation for 24 h using EdU assay, *n*=3. Scale bar: 25 μm. ***P* < 0.01 vs Control.


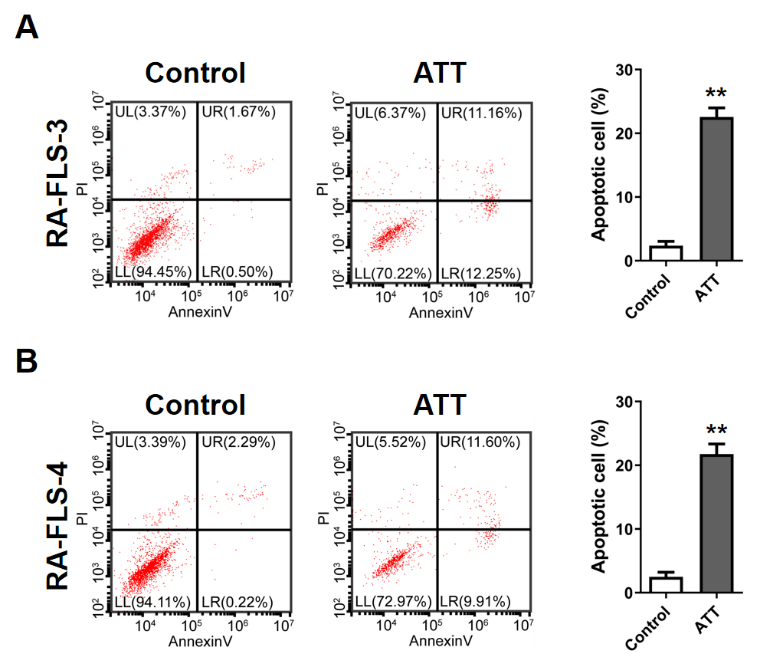


**Figure S5** ATT induces apoptosis of RA-FLSs. (A, B) Flow cytometry was adopted to estimate the apoptotic cells after ATT treatment for 24 h, *n*=3. ***P* < 0.01 vs Control.


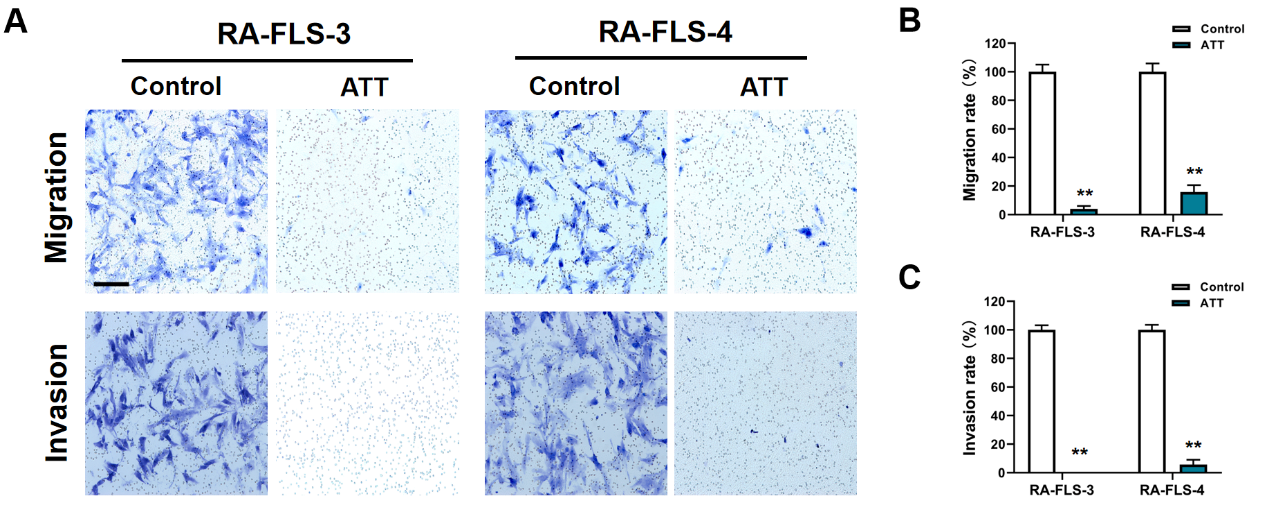


**Figure S6** ATT inhibits migration and invasion of RA-FLSs. RA-FLSs from 2 patients with RA were treated with ATT (10 µM) for 24 h. (A-C) Representative photographs of migrant and invasive cells were shown, and the statistical differences were measured after ATT stimulation, *n*=3. Scale bar: 50 μm. ***P* < 0.01 vs Control.

**
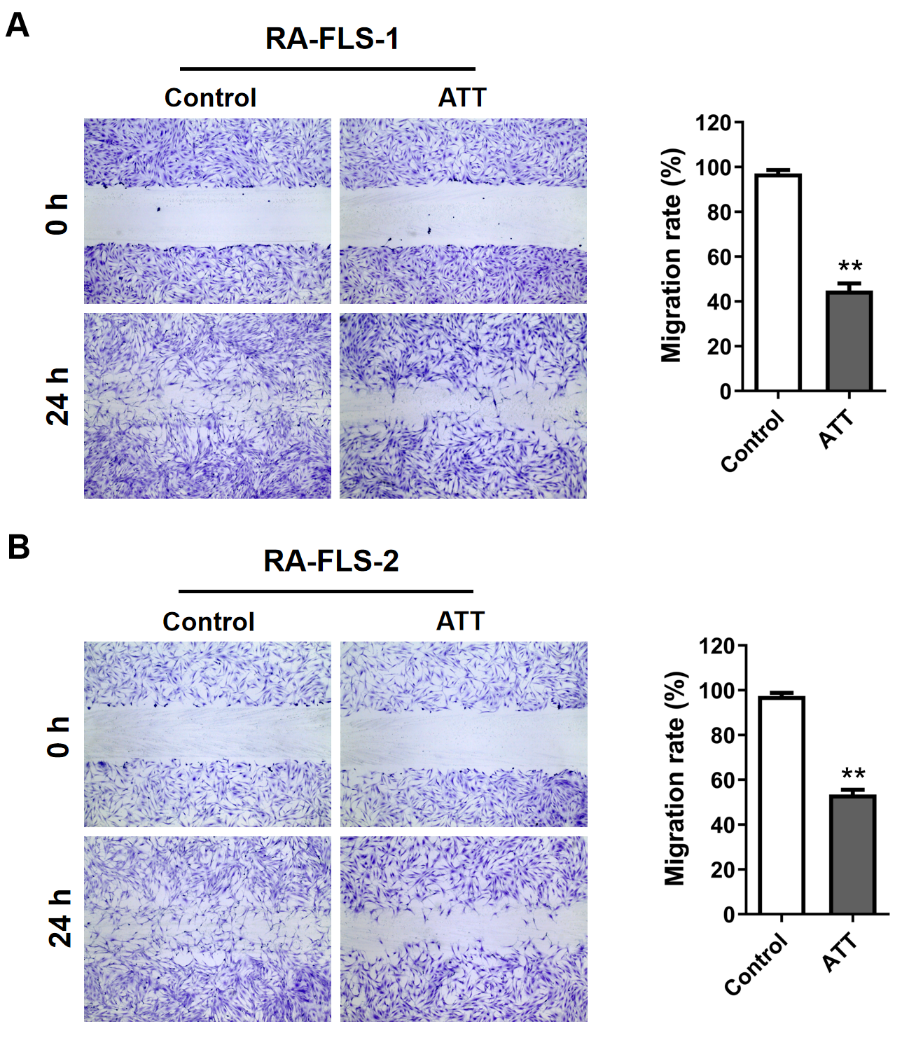
**

**Figure S7** ATT inhibits migration of RA-FLSs. RA-FLSs from 2 patients with RA were treated with ATT (10 µM) for 24 h, and the effect of ATT on cell migration was detected by scratch assay. (A-B) Representative photographs of migrant cells were shown, and the statistical differences were measured after ATT stimulation, *n*=3. ***P* < 0.01 vs Control.

**
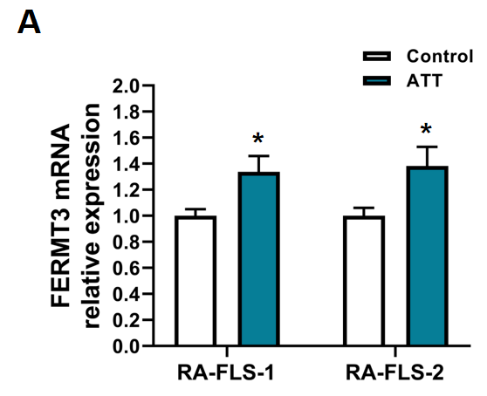
**

**Figure S8** Effect of ATT on the mRNA expression of FERMT3 in RA-FLSs. (A) The relative mRNA expression of FERMT3 after ATT (10 µM) treatment for 24 h in RA-FLSs were measured by RT-qPCR, *n*=3. **P* < 0.05 vs Control.


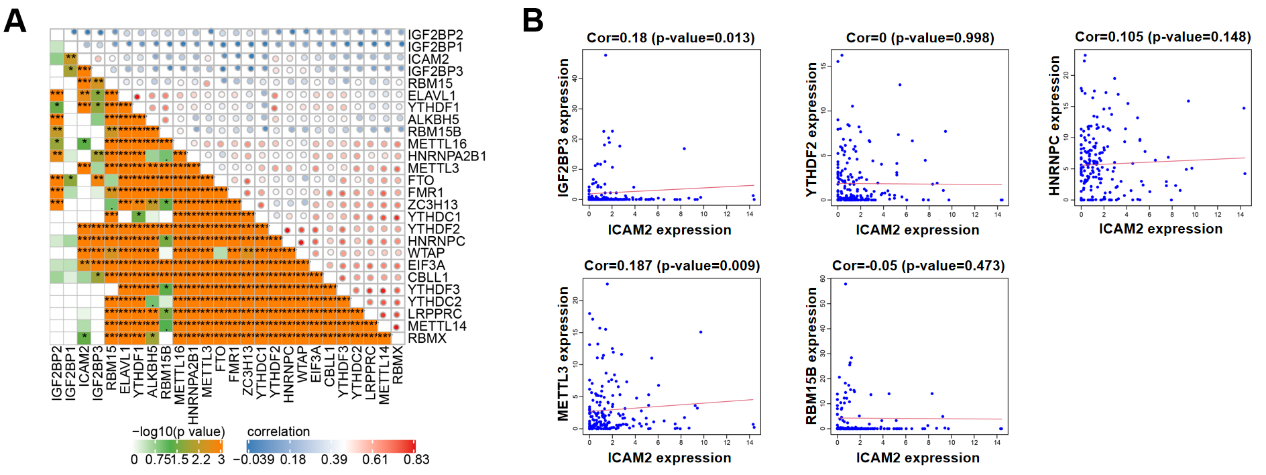


**Figure S9** Expression relationship of ICAM2 and METTL3. (A) RNA seq data obtained from the GEO datasets GSE89408 showing the expression relationship of ICAM2 and m6A regulators. (B) RNA seq data obtained from the GEO datasets GSE109449 showing the expression relationship of ICAM2 and IGF2BP3, YTHDF2, HNRNPC, METTL3, as well as RBM15B.


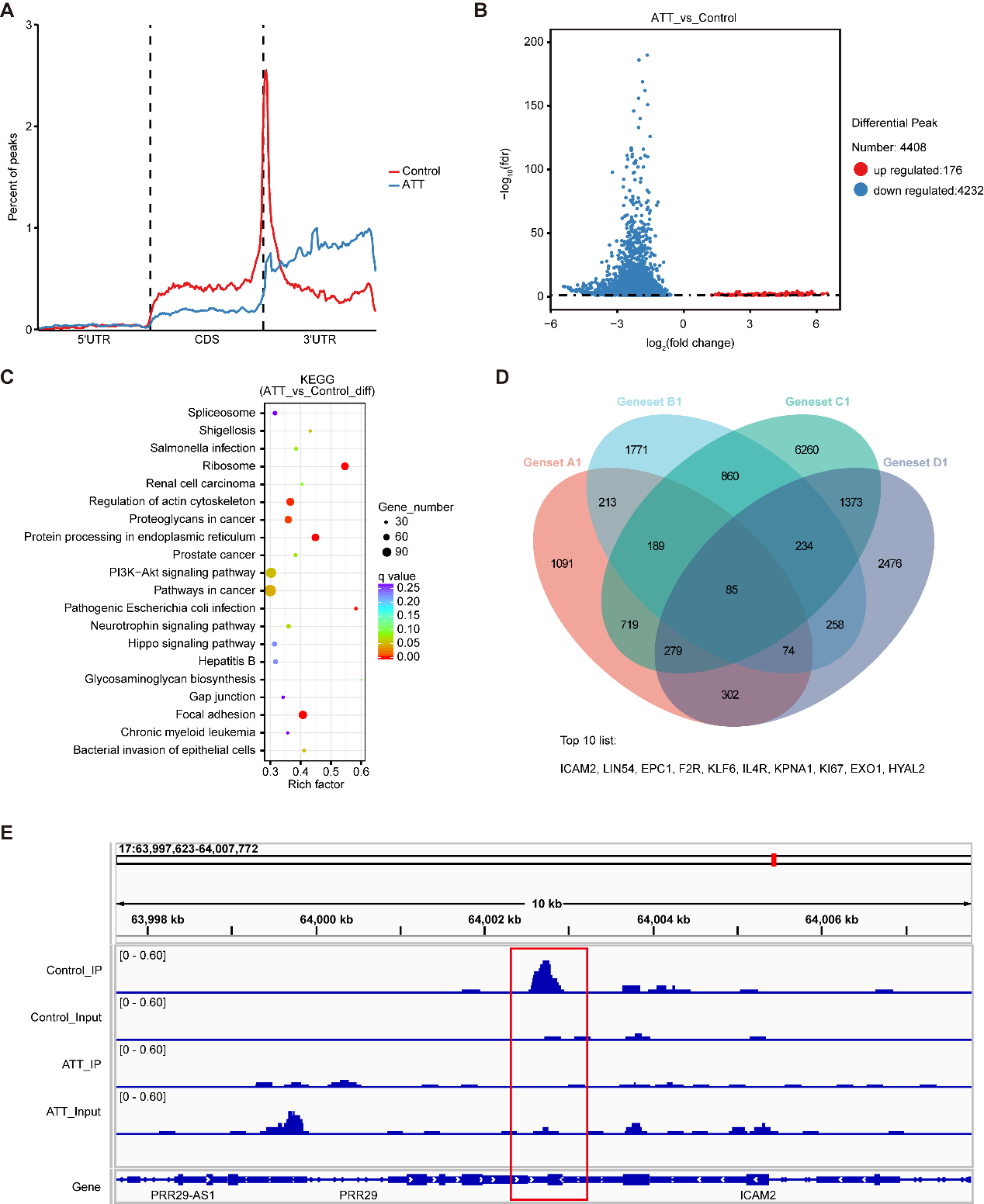


**Figure S10** The differential analyses of m6A-seq data in RA-FLSs. (A) The global view of the transcriptional region with the m6A peaks in ATT-treated RA-FLSs and the controls. (B) Volcano Plot displaying the differential m6A methylation peaks between ATT-treated RA-FLSs and the controls. (C) KEGG enrichment analyses showing the signals that the genes with differential m6A methylation after ATT treatment participated in. (D) Venn diagram presenting the intersection of genes in the 2952 genes with differential m6A methylation after ATT treatment (Geneset A1), the 3684 DEGs after ATT stimulation (Geneset B1), the 11980 genes correlated with RA in PEAC database (https://peac.hpc.qmul.ac.uk/) (Geneset C1), and 5081 genes related to RA in Genecards database (https://www.genecards.org/) (Geneset D1). (E) The m6A-seq data showing the m6A peak of *ICAM2* mRNA in ATT-treated RA-FLSs and the controls.


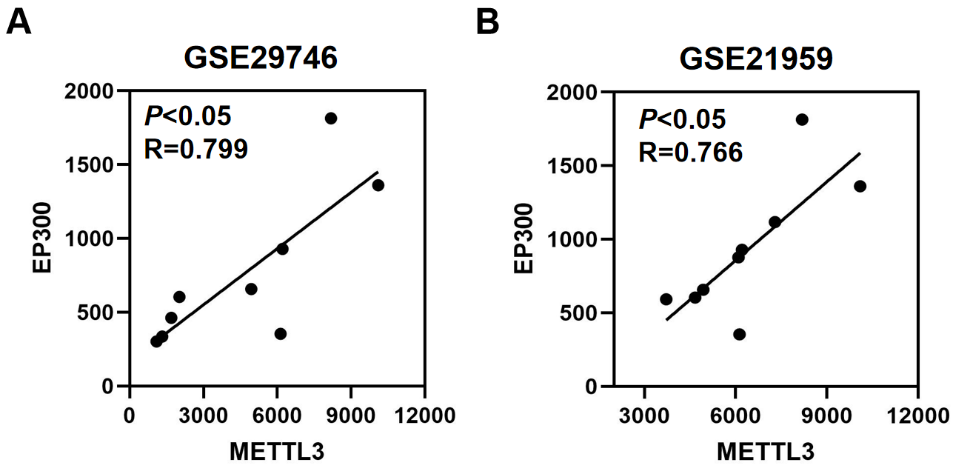


**Figure S11** The relationship between METTL3 and EP300 (p300). (A, B) RNA seq data obtained from the GEO datasets GSE29746 (A) and GSE21959 (B) showing that METTL3 has a strong positive correlation with EP300.


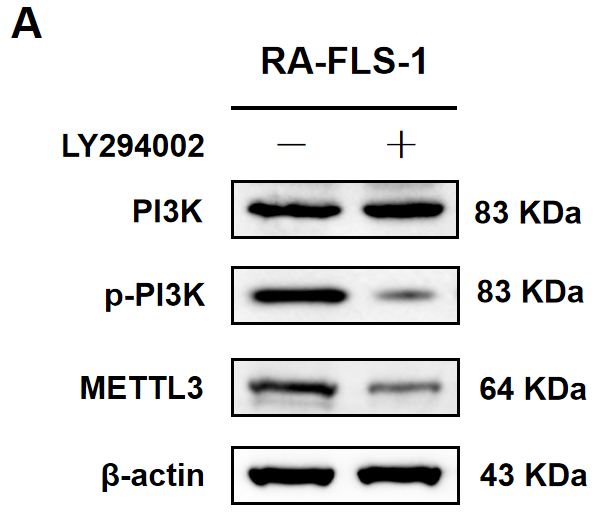


**Figure S12** Effect of LY294002 on the expression of METTL3 in RA-FLSs. (A) The protein expression of PI3K, p-PI3K, and METTL3 after LY294002 (10 µM) treatment for 24 h in RA-FLSs were measured by Western blot.


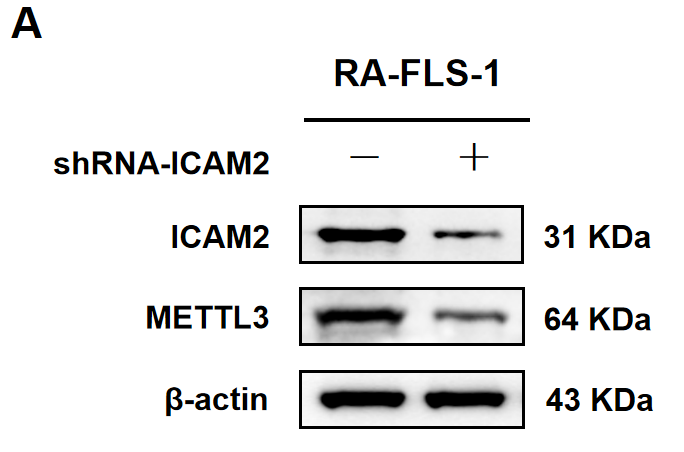


**Figure S13** Expression of METTL3 after knockdown of ICAM2 in RA-FLSs. (A) The protein expression of ICAM2 and METTL3 after knockdown of ICAM2 in RA-FLSs were measured by Western blot.


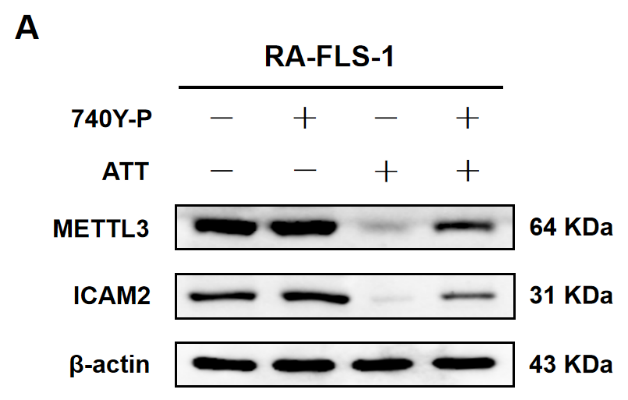


**Figure S14** 740Y-P reverses the expression of METTL3 and ICAM2 reduced by ATT in RA-FLSs. (A) The protein expression of METTL3 and ICAM2 after ATT stimulation (10 µM) for 24 h in RA-FLSs with or without 740Y-P in RA-FLSs were measured by Western blot.


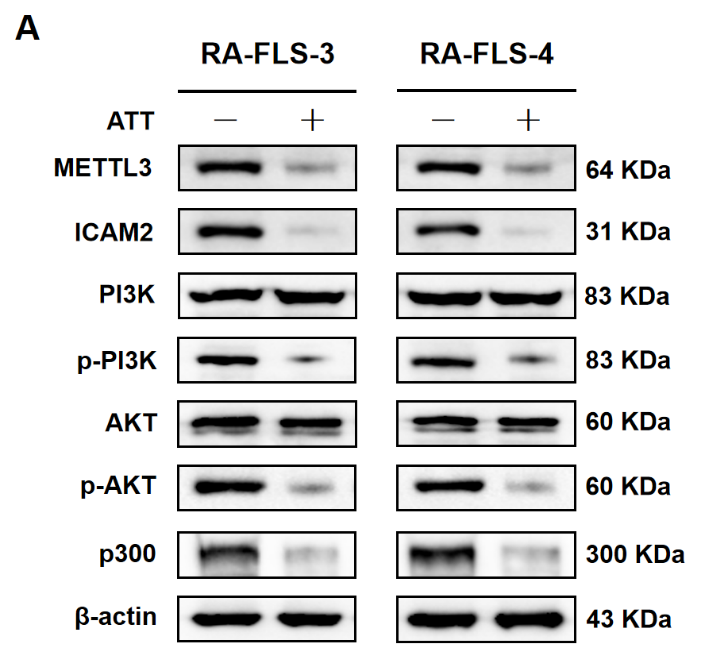


**Figure S15** ATT regulates the METTL3/ICAM2/PI3K/AKT/p300 pathway in RA-FLSs. (A) The protein expression of METTL3, ICAM2, PI3K, p-PI3K, AKT, p-AKT, and p300 after ATT (10 µM) treatment for 24 h in RA-FLSs were measured by Western blot.


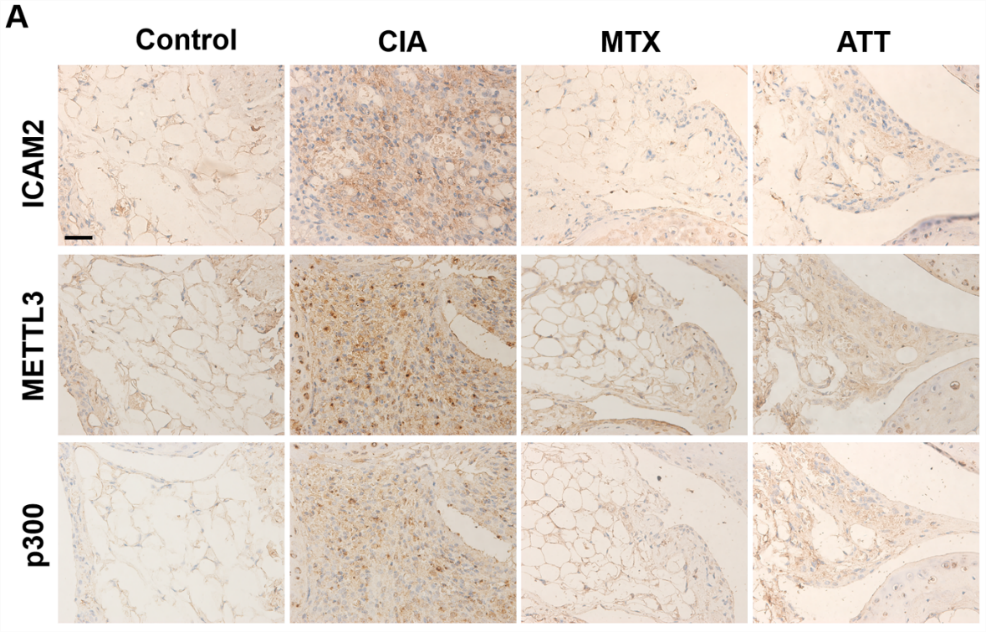


**Figure S16** Effect of ATT on the expression of ICAM2, METTL3, and p300 in synovial tissue of CIA mice. (A) Immunohistochemistry (IHC) staining showing ICAM2, METTL3, and p300 of paw sections after ATT treatment for indicated days. Scale bar: 12.5 μm.


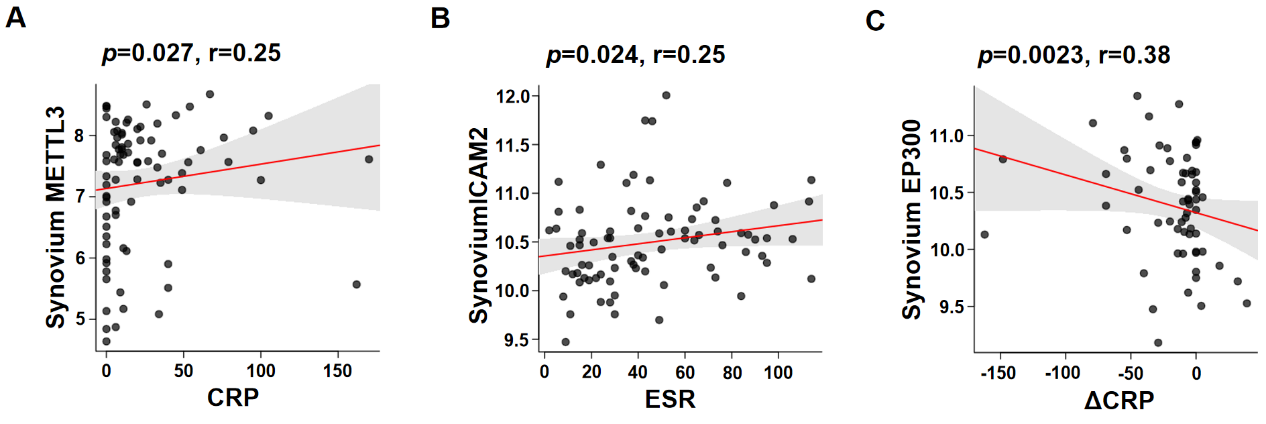


**Figure S17** The relationship between METTL3, ICAM2 and p300 expression in RA synovium tissues and the clinical characteristics and therapy response of RA patients. (A-C) The PEAC RNA-seq database from clinical specimens showed that METTL3 (A), ICAM2 (B) and p300 (C) expression in RA synovium tissues were correlated with clinical characteristics and therapy response of RA patients. CRP: C-reactive protein; ESR: erythrocyte sedimentation rate; ΔCRP: delta C-reactive protein.
